# Is a Lower Dose of Rivaroxaban Required for Asians? A Systematic Review of a Population Pharmacokinetics and Pharmacodynamics Analysis of Rivaroxaban

**DOI:** 10.3390/pharmaceutics15020588

**Published:** 2023-02-09

**Authors:** Xiao-Qin Liu, Zi-Ran Li, Chen-Yu Wang, Yue-Ting Chen, Zheng Jiao

**Affiliations:** 1 Department of Pharmacy, Shanghai Chest Hospital, Shanghai Jiao Tong University, Shanghai 200030, China; 2 Department of Pharmacy, Huashan Hospital, Fudan University, Shanghai 200437, China

**Keywords:** rivaroxaban, pharmacokinetics/pharmacodynamics, population pharmacokinetics, ethnic difference, Asians

## Abstract

Rivaroxaban has been widely used to prevent and treat various thromboembolic diseases for more than a decade. However, whether a lower dose of rivaroxaban is required for Asians is still debatable. This review aimed to explore the potential ethnic difference in pharmacokinetic/pharmacodynamic (PK/PD) characteristics between Asians and Caucasians. A systematic search was conducted and twenty-four studies were identified, of which 10 were conducted on Asian adults, 11 on predominantly Caucasian adults, and 3 on Caucasian pediatrics. The apparent clearance (CL/F) of rivaroxaban in Caucasian adults with non-valvular atrial fibrillation (6.45–7.64 L/h) was about 31–43% higher than that in Asians (4.46–5.98 L/h) taking 10~20 mg rivaroxaban every 24 h. Moreover, there was no obvious difference in CL/F among Japanese, Chinese, Thai, and Irani people. Regarding PK/PD relationship, prothrombin time was linked to rivaroxaban concentration in a linear or near-linear manner, and Factor Xa activity was linked with the E_max_ model. The exposure–response relationship was comparable between Asians and Caucasians. Renal function has a significant influence on CL/F, and no covariate was recognized for exposure–response relationship. In conclusion, a lower dose of rivaroxaban might be required for Asians, and further studies are warranted to verify this ethnic difference to facilitate optimal dosing regimens.

## 1. Introduction

Rivaroxaban is one of the most commonly used direct oral anticoagulants (DOACs) for the management of several thromboembolic disorders, such as deep vein thrombosis, pulmonary embolism, non-valvular atrial fibrillation (NVAF), and acute coronary syndrome (ACS) [1]. It has also been approved for the treatment and reduction of the recurrence risk of venous thromboembolism (VTE) in children [2,3].

Following oral administration, rivaroxaban is rapidly absorbed and reaches a peak concentration within 2–4 h [4]. The bioavailability of rivaroxaban is dose-dependent, reaching 80–100%, without being affected by food, upon the oral administration of 2.5–10 mg tablets. However, bioavailability is decreased by 34% in healthy subjects when it is administered as 20 mg tablets under fasting conditions [4,5]. The plasma protein binding for rivaroxaban is approximately 92–95%. Further, the clearance (CL) of rivaroxaban is a dual pathway: approximately 2/3 of rivaroxaban is metabolized by hepatic cytochrome P450 (CYP) enzymes 3A4/5, 2J2, and CYP-independent enzymes, while the remaining 1/3 is eliminated unchanged via the kidney, involving transporters in active renal secretion such as P-glycoprotein (P-pg) and breast cancer resistance protein (BCRP) [4,6]. The anticoagulant effect of rivaroxaban is regarded as the direct inhibition of free and clot-bound Factor Xa (FXa). Rivaroxaban could also prolong prothrombin time (PT) and activated partial thromboplastin time (aPTT) [4,7].

A dosing regimen of 20 mg q24h was approved for patients with NVAF according to global clinical trials [8]. Meanwhile, a dosing regimen of 15 mg q24h was approved for Japanese patients [9]. Although the recommended dosing regimen for other Asians with NVAF, such as Chinese, South Korean, and Thai patients, was determined as 20 mg q24h, some studies recently found that a dose lower than that recommended might have the same efficacy and be safer for Asians [10,11,12]. Although rivaroxaban has been widely used for more than 10 years worldwide, the factors that account for this discrepancy remain unknown [13]. Moreover, whether Asians actually require a lower dose of rivaroxaban, compared to that for Caucasians, to achieve similar efficacy and safety is still debatable.

Some factors were assessed in terms of whether they influence the pharmacokinetics (PK) and pharmacodynamics (PD) of rivaroxaban during clinical development. Only renal function was found to have a notable impact on the CL of rivaroxaban, and dose regimens in patients with NVAF should be adjusted accordingly [8]. Because patients in the real world could be different from those treated during clinical trials, it is not clear whether there are any other factors affecting the PK/PD of rivaroxaban. 

The population approach is widely used to identify the pathophysiological factors that cause changes in the dose–exposure–response relationship and, accordingly, if dose adjustment is required. Terrier et al. [14] reviewed the effect of covariates on exposure to rivaroxaban [14], but they did not assess the difference in PK/PD among various ethnicities. Moreover, several population PK and PK/PD studies in Asians were reported to identify significant covariates based on CL in recent years. Thus, this review aimed to (i) explore the potential ethnic differences in the PK/PD of rivaroxaban between Asian and Caucasian populations and (ii) summarize the covariates affecting the PK/PD of rivaroxaban.

## 2. Methods

### 2.1. Study Identification

Population PK and PK/PD studies of rivaroxaban were systematically searched in the PubMed, Embase, and Web of Science databases from inception through 31 August 2022. The following search terms were employed: “rivaroxaban”, and “population pharmacokinetic”, or “pharmacokinetic model*”, “pharmacokinetic and pharmacodynamic”, “pharmacokinetic/pharmacodynamic”, “pharmacokinetic-pharmacodynamic”, “nonlinear mixed effect”, “NONMEM”, “WinNonMix”, “MONOLIX”, “Pmetrics”, “ADAPT”, “P-PHARM”, “nlmixed”, “NLME”, or “USC PACK.” The reference lists of the included articles were also checked. A literature search was performed by two independent authors and inspected by a third author.

All published population PK and PK/PD models of rivaroxaban were included if they met the following criteria: (i) study population: patients or healthy subjects, (ii) treatment with rivaroxaban as the studied drug, and (iii) data analysis: population PK or population PK/PD analysis.

Articles were excluded if they met the following criteria: (i) they were reviews, conference abstracts, or focused on methodology/algorithm/software, (ii) they were not published in the English language, or (iii) they did not provide sufficient information on the methodology and population PK or population PK/PD model.

### 2.2. Reporting Quality

According to the previous guidelines established by Kanji et al. [15] and Jamsen et al. [16], a 30-item checklist was developed to evaluate literature quality, with information to be included when reporting a transparent and accurate clinical PK/PD study. If an item in the checklist was reported in the study, 1 point was counted in the scoring item; otherwise, no point was counted. The total score for each study was calculated and shown as a percentage, which was defined as the compliance rate.

### 2.3. Data Extraction

The following information was extracted from the identified articles: (i) characteristics and demographics of the study population (e.g., patients/healthy subjects, age, body weight (BW), sex, and renal and hepatic function); (ii) study design (e.g., study type, number of included subjects, dosing regimens, sampling design, and bioassay method used); and (iii) information on population PK or population PK/PD analyses (e.g., structural model, statistical model, parameter estimates, covariates, model evaluation approaches, and model application).

### 2.4. Comparison of Studies

The study characteristics, population PK, and population PK/PD analyses were summarized in a tabular format. The visual predictive distributions (VPDs) of the concentration-time and PD biomarker-time profiles at steady state were generated via Monte Carlo simulations based on reported population PK/PD models from each study [17]. A total of 1000 virtual patients were simulated for each scenario. All simulations were performed using NONMEM software (version 7.5; ICON Development Solutions, Ellicott City, MD, USA).

The effects of the included covariates on the PK parameters and PD metrics were assessed using forest plots. Continuous covariates, such as age, BW, and serum creatinine (SCr) level, were scaled to the same range. In contrast, binary covariates such as sex were expressed as 0 and 1. The upper and lower limits of the parameters were estimated based on the range of the corresponding covariates. Each range was normalized to the median value in each model. Therefore, the effect of each covariate can be shown as a range of the limit to the median value, as follows (Equation (1)):

(1) $$\mathrm{effect}\;\mathrm{of}\;\mathrm{covariate}\;(i)\;\mathrm{in}\;\mathrm{model}\;(j)=\frac{estimated\;range\;of\;parameter\;\left(k\right)}{median\;value\;of\;parameter\;\left(k\right)\;in\;model\;\left(j\right)}\times 100\%$$

Data were analyzed and plotted using R software (version 4.2.1; www.r-project.org; accessed on 1 September 2022).

## 3. Results

### 3.1. Study Identification

A total of 353 publications were initially selected from the PubMed, EMBASE, and Web of Science databases, of which 167 were excluded after screening. After a full-text review, 23 studies were deemed eligible. One additional study was identified from the reference list of the included studies [18]; hence, a total of 24 articles published between 2007 and 2022 were retained. A PRISMA diagram is shown in Figure S1.

### 3.2. Reporting Quality

The 30-item checklist and corresponding analysis outcomes of each study are shown in Table S1. The range of compliance in the included studies was 43.3–93.3%, with a median compliance of 80.8%. The compliance in 18 of the 24 studies was greater than or equal to 80%. Moreover, there was no obvious difference in compliance between studies published before or after publishing the guidelines [15,16] (81.7% vs. 80.3%).

### 3.3. Study Characteristics

#### 3.3.1. Basic Characteristics

All 24 studies included were prospective, and each of their characteristics is listed in Table 1. Two studies involved healthy adult subjects [19,20], nineteen studies included adult patients [18,21,22,23,24,25,26,27,28,29,30,31,32,33,34,35,36,37,38], and three studies were conducted on pediatric patients [39,40,41]. Ten studies (41.7%) were from the Asians, including Japanese (*n* = 4), Chinese (*n* = 4), Thai (*n* = 1), and Iranian population (*n* = 1), the study cohorts of which were almost all patients with NVAF [18,20,25,26,30,34,35,36,37,38]. Fourteen studies were conducted on population of multiple ethnicities, which were all or predominantly Caucasians [19,21,22,23,24,27,28,29,31,32,33,39,40,41]. Twelve studies (50%) developed a model with data from phase I, II, or III clinical trials [19,21,22,23,24,25,26,27,29,31,39,40,41], while the remaining studies enrolled subjects from real-world clinical settings [18,20,28,30,32,33,34,35,36,37,38].

Zhang et al. [29] combined data from the studies by Mueck et al. [23] and Girgis et al. [27]. Willmann et al. pooled data from five previous studies by Mueck et al. [21,22,23], Xu et al. [24], and Girgis et al. [27]. The study by Speed et al. [33] applied the data obtained in study by Barsam et al. [28] and enrolled additional patients to ensure a greater sample set.

In most studies, population analysis was performed using the NONMEM software. Two used Phoenix NLME software [18,30], while one study used Monolix software [38].

#### 3.3.2. Population Pharmacokinetic Models

The final population PK parameters of the included studies are presented in Table 2. Nineteen studies (79.2%) described the PK of rivaroxaban using a one-compartment model, whereas the other five used a two-compartment model [19,20,39,40,41]. Twenty-two studies (91.7%) described the process of first-order absorption and elimination, while one study employed sequential zero-order and first-order absorption [20], and another study employed the lag time on absorption [19]. The plasma concentration of rivaroxaban was measured using high-performance liquid chromatography with tandem mass spectrometry (*n* = 19) [19,20,21,22,23,24,25,26,27,29,30,31,34,35,36,37,39,40,41] or anti-Xa assays (*n* = 5) [18,28,32,33,35].

#### 3.3.3. Population Pharmacokinetic-Pharmacodynamic Models

Thirteen studies developed population PK/PD models of rivaroxaban, which linked the plasma concentration of rivaroxaban to response, including parameters such as PT (*n* = 13) [19,20,21,22,23,24,25,26,27,30,32,34,38], FXa activity (*n* = 4) [19,22,26,27], anti-Xa activity (*n* = 2) [20,38], aPTT (*n* = 4) [19,26,32,38], Heptest (*n* = 2) [19,26], and prothrombinase-induced clotting time (PiCT, *n* = 1) [27].

PT was measured using various thromboplastin reagents in different studies. It was correlated with rivaroxaban concentration in either a linear (Equations (2) and (3)) or near-linear relationship (Equations (4) and (5)):

(2) $$M=M_{0}+slope\times C_{p}$$

(3) $$M=M_{0}+slope\times \log(C_{p})$$

(4) $$M=M_{0}+slope\times C_{p}^{1-Hill\times C_{p}}$$

(5) $$M=M_{0}+slope\times C_{p}^{Hill}$$

where *M* represents the measurement of PT (APTT, Heptest, or PiCT), *M*_0_ represents the baseline of PT (APTT, Heptest, or PiCT), *slope* represents the change in PT (APTT, Heptest, or PiCT) per unit rivaroxaban concentration change, *C_p_* represents the plasma concentration of rivaroxaban, and *Hill* represents the exponent of rivaroxaban concentration.

FXa activity was measured directly using a two-step photometric assay [19,22,26] or expressed as a percentage change compared with the control plasma [27]. It was linked to the rivaroxaban concentration with an E_max_ or sigmoid E_max_ model, as shown in Equations (6)–(8):

**Table 1:** Study characteristics.

Study (Year)	Study Type	Country/Race	Study Population	No. of Subjects (M/F)	No. of Samples (Per Person)	Age (Years) ^a^	Body weight (kg) ^a^	Lean body mass (kg) ^a^	CrCl (or eGFR, mL/min) ^a^	Dose Regimens
Mueck et al. (2007) [19]	Phase I	Caucasian	Healthy subjects	43 (43/0)	PK: 1809 (42.1) PD markers in total: 6533 (151.9) ^b^	32.5 [20–45]	NA	NA	NA	5 mg qd, 5–30 mg bid, 5 mg tid
Mueck et al. (2008a) [21]	Phase II	Europeans, Israeli	HRS	PK: 758 (302/456) PT: 1118 (NA)	PK: 5743 (7.6) PT: 10467 (8.9)	66 [26–93] ^c^	75 [45–120] ^c^	NA	88.1 [18.8–208]	2.5–30 mg bid, 5–40 mg qd
Mueck et al. (2008b) [22]	Phase II	Canadians, American, Europeans	HRS, KRS	1013 (NA)	PK: 7660 (7.6) PD markers in total: >9100 (>9.0) ^d^	HRS: 65 [26–87] KRS: 67 [39–92]	HRS: 76 [45–125] KRS: 86 [50–173]	HRS:51 [34–81] KRS: 51 [28–83]	HRS: 96 [33–218] KRS: 104 [35–259]	2.5–30 mg bid
Mueck et al. (2011) [23]	Phase II	Most Caucasian	DVT	870 (487/383)	PK: 4634 (5.3)	61 [18–94]	NA	Male: 63 ± 8 Female: 47 ± 5	87.4 ± 1.5	10–30 mg bid, 20–40 mg qd
Xu et al. (2012) [24]	Phase III	White (95.8), Black (0.8%), Asian (1.7%), Others (1.7%)	ACS	2290 (1784/506)	PK: NA PT: 6644 (4.9) ^e^	57 [24–87]	84 [36–181]	60.7 [30.4–90.4]	96.9 [22.4–298]	2.5–10 mg bid, 5–20 mg qd
Tanigawa et al. (2013) [26]	Phase II	Japanese	NVAF	182 (148/34)	PK: 842 (4.6) FXa activity: 985 (5.4) PT: 987 (5.4) aPTT: 986 (5.4) Heptest: 987 (5.4)	65.6 ± 10.0 [30–92]	67.2 ± 10.4 [45–103]	51.4 ± 7.2 [34.7–67.9]	Baseline: 79.7 ± 25.2 [29.0–175.8] Day 28: 80.7 ± 26.6 [29.0–198.8]	10–20 mg qd, 2.5–20 mg bid
Kaneko et al. (2013) [25]	Phase III	Japanese	NVAF	597 (NA)	PK: 1834 (3.1) PT: 1869 (3.1)	70.98 ± 8.31 72 [34–89]	64.45 ± 10.65 63.9 [35–104]	49.69 ± 7.14 50.24 [30.18–70.48]	67.41 ± 22.89 64 [26–172]	15 mg qd (10 mg qd in patients with CrCl < 50 mL/min)
Girgis et al. (2014) [27]	Phase III	Most Caucasian	NVAF	161 (NA)	PK: 801 (5.0) FXa activity: 799 (5.0) PT: 796 (5.0) PiCT: 742 (4.6)	65 ± 9.5	NA	57.5 ± 9.9	NA	20 mg qd (15 mg qd for patients with CrCl 30–49 mL/min)
Zhang et al. (2017) [29]	Phase II, III	Most Caucasian	DVT, NVAF	285 (NA)	NA	NVAF: 65 [51–81] ^f^ DVT: 59 [31–83] ^f^	NA	NVAF: 56.6 [42.5–73.6] ^f^ DVT: 54.1 [40.1–72.7] ^f^	NA	20 mg qd (15 mg for NVAF patients with CrCl 30–49 mL/min)
Barsam et al. (2017) [28]	Post- marketing study	Caucasian (74%), Afro-Caribbean (21%), Other (5%)	Acute VTE treatment, VTE prevention	101 (59/42)	PK: 193 (1.9) ^g^	52 [20–86] ^h^	88 ± 23.4	57.0 ± 11.3	67%: > 80 mL/min 25%: 50–79 mL/min 7.8%: 30–49 mL/min 0.2%: < 30 mL/min	10–20 mg qd, 15 mg bid
Suzuki et al. (2018) [30]	Post- marketing study	Japanese	NVAF	96 (81/15)	PK: 192 (2) PT: 192 (2)	68.0 ± 9.5	69.1 ± 11.4	NA	Baseline: 76.2 ± 21.3 2–4 h after drug intake: 77.6 ± 21.8	15 mg qd (10 mg in patients with CrCl < 50 mL/min)
Willmann et al. (2018a) [31]	Phase II, III	Multinational	HRS, KRS, DVT, ACS, NVAF	4918 (2985/1933)	PK: 22843 (4.6)	60.53 ± 11.82	82.48 ± 16.87	57.05 ± 10.00	97.74 ± 33.97 ^i^	2.5–30 mg bid, 2.5–40 mg qd
Willmann et al. (2018b) [39]	Phase I	White (74.6%), Black (1.7%), Asian (3.4%), Hispanic (11.9%), Missing (6.8%)	Children with VTE	59 (33/26)	PK: 206 (3.5)	6.8 ± 4.9 6.0 [0.5–17]	29.5 ± 18.3 27.7 [6.2–77.8]	NA	NA	10, 20 mg
Zdovc et al. (2019) [32]	Post- marketing study	Slovenia	HRS, KRS	17 (8/9)	PK: 82 (4.8) ^g^ PT: NA aPTT: NA	64 [49–82]	84 [54–125]	53 [38–81]	82 [57–150] ^j^	10 mg qd
Goto et al. (2020) [18]	Post- marketing study	Japanese	NVAF	119 (85/34)	PK: 162 (1.3) ^g^	10 mg: 73.1 ± 10.0 15 mg: 66.7 ± 10.0	10 mg: 60.3 ± 15.5 15 mg: 67.3 ± 13.8	NA	10 mg: 64.0 ± 21.2 15 mg: 84.4 ± 27.7	10, 15 mg qd
Speed et al. (2020) [33]	Post- marketing study	The United Kingdom	VTE, NVAF, other	913 (522/391)	PK: 1108 (1.2) ^g^	67.03 ± 15.00 [19–96]	85.75 ± 23.07 [39–172]	55.8 ± 13.1	91.47 ± 43.81 [16–259]	10–30 mg qd, 10–15 mg bid
Willmann et al. (2021) [40]	Phase III	Most Caucasian	Children with VTE, post- Fontan surgery	524 (NA)	PK: 1988 (3.8)	Not defined (16% <2 years)	<6 months: 4.1 ± 1.3 [2.7–7.9] 6 months–2 years: 9.5 ± 2.3 [5.4–15.1] 2–6 years: 16.4 ± 4.2 [10.1–39.1] 6–12 years: 32.4 ± 10.8 [17–71] 12–18 years: 67.9 ± 21.1 [20–194]	NA	<6 months: 111 ± 45.6 [50.6–220] 6 month–2 years: 156 ± 52.4 [74.4–456] 2–6 years: 168 ± 49.4 [43.8–287] 6–12 years: 178 ± 43.5 [76.7–330] 12–18 years: 144 ± 38.3 [73.8–354]	0.4–20 mg qd
Esmaeili et al. (2022) [38]	Post- marketing study	Iranian	NVAF, DVT, PE	69 (33/36)	PK: 126 (1.8)	64 [36–86]	70 [46–112]	49.6 [34.8–72.0]	63.8 [23.5–128.5]	20 mg bid, 10–20 mg qd
Liu et al. (2022) [34]	Post- marketing study	Chinese	NVAF	195 (111/84)	PK: 256 (1.3) PT: 244 (1.3)	66.7 ± 11.7 68 [28–96]	68.9 ± 12.9 68 [36.5–119]	51.5 ± 8.9 51.8 [29.0–73.3]	77.8 ± 21.2 79.7 [21.9–127.7]	5–20 mg qd
Singkham et al. (2022) [35]	Post- marketing study	Thai	NVAF	60 (38/22)	PK: 240 (4) ^g^	69.4 ± 9.2	64.0 ± 14.1	NA	59.0 ± 22.8	10–20 mg qd
Willmann et al. (2022) [41]	Phase III	Multinational	Children with congenital heart disease and undergone the Fontan procedure	NA	NA	NA	NA	NA	NA	NA
Zhang et al. (2022) [37]	Post- marketing study	Chinese	NVAF	150 (95/55)	PK: 263 (1.8)	68.0 ± 12.6 69 [23–90]	67.4 ± 20.8 66.1 [38.0–110.0]	NA	72.1 ± 33.5 71.1 [13.3–146.7]	10,15, 20 mg, others
Zhao et al. (2022) [20]	Post- marketing study	Chinese	Healthy subjects, NVAF	Healthy subjects: 304 (202/102) NVAF: 223 (118/105)	PK: 4726 (15.5) ^k^ PT: 1624 (3.1) Anti-Xa: 1253 (2.4)	Healthy subjects: 30 [18–62] NVAF: 70 [34–91]	Healthy subjects: 62.8 [47.0–83.0] NVAF: 68.2 [38–112]	NA	Healthy subjects: 103 [71.5–568] NVAF: 76.3 [26.1–178]	Healthy subjects: 10–20 mg (single dose) NVAF: 5–30 mg qd
Zhang et al. (2023) [36]	Post- marketing study	Chinese	NVAF	180 (96/84)	PK: 360 (2)	81.8 ± 4.3 81 (75–95)	67.3 ± 12.4 70 (40–100)	NA	71.7 ± 22.1 73.3 (20.4–113.7)	5–20 mg qd

Abbreviations: ACS: patients with acute coronary syndrome; aPTT: activated partial thromboplastin time; CrCl: creatinine clearance; DVT: patients with deep vein thrombosis; eGFR: estimated glomerular filtration rate; FXa: Factor Xa; HRS: patients with hip replacement surgery; KRS: patients with knee replacement surgery; NA: not available; NVAF: patients with non-valvular atrial fibrillation; PD: pharmacodynamics; PE: patients with pulmonary embolism; PiCT: prothrombinase-induced clotting time; PK: pharmacokinetics; PT: prothrombin time; VTE: patients with venous thrombus embolism. ^a^ the data were shown as mean ± SD or median [min–max]. ^b^ PD data included FXa activity, PT, aPTT, and Heptest. ^c^ Only data from PK cohorts were provided. ^d^ PD data include FXa activity and PT. ^e^ PT data was from 1347 patients. ^f^ The data was shown as median [5th percentile–95th percentile]. ^g^ PK data was measured by anti-Xa assays. ^h^ the data was shown as mean [min–max]. ^i^ CrCl was calculated using modified Crockroft–Gault equation. ^j^ eGFR calculated using modified diet in renal disease equation. ^k^ The PK data were only obtained from healthy subjects.

**Table 2:** Final population pharmacokinetic parameters of included studies.

Study (Year)	Estimation Method	Fixed Effect Parameters		Between-Subject Variability	Residual Unexplained Variability	Model Evaluation	Model Application
Mueck et al. (2007) [19]	FOCE	*k*_a_ (/h)	0.97	52.9% (BSV) 93.8% (IOV)	25.4%	GOF, OFV	Determination of sampling window in phase II
		ALAG (h)	0.25	102.5% (IOV)			
		CL/F (L/h)	9.17	17.4%			
		V_c_/F (L)	55.3 (dose < 30 mg) 79.2 (dose = 30 mg)	30.7%			
		V_p_/F (L)	12.6 (dose < 30 mg) 23.5 (dose = 30 mg)	38.6%			
		Q/F (L/h)	1.35	/			
Mueck et al. (2008a) [21]	FOCE-I	*k*_a_ (/h)	0.047 (day 2 for mixed population 1) 0.222 (day 3–4 for mixed population 1) 1.07 (≤ 4 days for mixed population 2) 1.49 (> 4 days for all patients)	/	52.6%	GOF, OFV	NA
		CL (L/h)	5.46 (day 2) 6.91 (day 3–4) 7.51 × [1 − 0.01 × (age − 66) + 0.003 × (CrCl − 88.1) + 0.22 × (ALB − 3.4) − 0.012 × (HCT − 37.3)] (day > 4)	38.2%			
		V (L)	58.2 × [1 + 0.64 × (BSA − 1.84)]	32.4%			
		F	0.877 (5, 10 mg, compared to 2.5 mg) 0.791 (20 mg, compared to 2.5 mg)	/			
Mueck et al. (2008b) [22]	FOCE-I	*k*_a_ (/h)	0.092 (≤ 3 days for population 1, HRS) 1.81 (≤ 3 days for population 2 and > 3 days for the total population, HRS) 1.20 (> 3 days, KRS)	/	37.1% (HRS) 34% (KRS)	GOF, OFV	NA
		CL (L/h)	6.4 (≤ 3 days, HRS) 7.3 × [1 − 0.015 × (age − 65) − 0.21 × (SCr − 0.78)] (> 3 days, HRS) 6.13 × [1 + 0.002 × (CrCl − 103)] × 0.85 (if female) (> 3 days, KRS) ^a^	70.1% (HRS, ≤ 3 days) 38.6% (HRS, > 3 days) ^b^			
		V (L)	49.1 × [1 + 0.018 × (LBM − 51)] (HRS) 55.7 × [1 + 0.67 × (BSA − 1.95)] (KRS)	/			
		F	1 (2.5 mg bid) 0.847 (HRS, 5, 10 mg bid) 0.74 (KRS, 5, 10 mg bid) 0.609 (HRS, 20, 30 mg bid) 0.53 (KRS, 20, 30 mg bid)	/			
Mueck et al. (2011) [23]	FOCE-I	*k*_a_ (/h)	1.23	/	40.7%	GOF, bootstrap, VPC	The effect of identified factor on PK, and factors influencing PK in NVAF patients
		CL (L/h)	5.67 × [1 − 0.007 × (age − 61) − 0.269 × (SCr − 0.94)]	39.9%			
		V (L)	54.4 × [1 + 0.008 × (LBM − 56) − 0.005 × (age − 61)]	28.8%			
		F	0.79 (20 mg compared to 10 mg) 0.63 (30, 40 mg compared to 10 mg)	/			
Xu et al. (2012) [24]	FOCE	*k*_a_ (/h)	1.24	139%	35.2 %	GOF, VPC	The effect of identified factors on PK
		CL (L/h)	6.48 × [1 − 0.0112 × (age − 57) − 0.151 × (SCr − 0.95)]	31.3% (BSV) 32.4% (IOV)			
		V (L)	57.9 × [1 + 0.00833 × (LBM − 60.7) − 0.00707 × (age-57)]	10.0%			
		F	1 (2.5 mg) 0.851 (< 10 mg) 0.705 (≥ 10 mg)	/			
Tanigawa et al. (2013) [26]	FOCE-I	*k*_a_ (/h)	0.6	68%	40.2%	GOF, VPC, bootstrap	Comparison of PK parameters in Japanese and Caucasian patients
		CL (L/h)	4.72 × [1 − 0.0165 × (BUN − 16.73)]	21.3%			
		V (L)	42.9	/			
		F	1	24.4%			
Kaneko et al. (2013) [25]	FOCE-I	*k*_a_ (/h)	0.617	58.2%	13.1%	GOF, VPC, bootstrap	Comparison of the posthoc estimated PK parameters between Japanese and non-Japanese patients
		CL (L/h) ^c^	4.73 × (CrCl/67.11)^0.159^ × [1 − 0.0132 × (HCT − 42.14)]	41.0%			
		V (L)	43.8	63.6%			
		F	1	37.7% (IOV)			
Girgis et al. (2014) [27]	FOCE-I	*k*_a_ (/h)	1.16	/	47.9%	PE%, GOF, pc-VPC	Dose modification for patients with renal impairment
		CL/F (L/h)	6.1 × [1 − 0.011 × (age − 65) − 0.194 × (SCr − 1.09)]	35.2%			
		V/F (L)	79.7 × [1 − 0.00133 × (age − 65) + 0.0118 × (LBM − 57.5)]	17.6%			
Zhang et al. (2017) [29]	FOCE-I	*k*_a_ (/h)	0.982	/	47.5%	GOF, VPC	Cross-study PK comparison
		CL/F (L/h)	6.31 × [1 − 0.011 × (age − 65) − 0.244 × (SCr − 1.05)]/1.12 (if DVT)	34.6%			
		V/F (L)	70.3 × [1 − 0.00347 × (age − 65) − 0.109 × (LBM − 56.62)]/1.12 (if DVT)	15.5%			
Barsam et al. (2017) [28]	FOCE	*k*_a_ (/h)	1.21	/	31% 0.016 ng/mL	VPC	NA
		CL/F (L/h)	8.86 × (CrCl/79)^0.434^	48%			
		V/F (L)	101	60%			
Suzuki et al. (2018) [30]	FOCE	*k*_a_ (/h)	1.37	44.6%	41.8%	GOF	NA
		CL/F (L/h)	4.40 × (CrCl/75)^0.324^ × (ALT/22)^−0.225^ × (1 − 0.319 × INH)	20.6%			
		V/F (L)	38.2	63.6%			
Willmann et al. (2018a) [31]	FOCE-I	*k*_a_ (/h)	0.821	79.25%	45.06%	GOF, pc-VPC	The effect of identified factors on PK
		CL (L/h)	6.58 × (CrCl/93)^0.406^ × (BW/81) ^−0.278^ × 0.966 (if co-medication with P-pg inhibitor) × 0.978 (if co-medication with strong CYP3A4 inhibitors) × 0.863 (if co-medication with moderate CYP3A4 inhibitors) × 0.939 (if co-medication with weak CYP3A4 inhibitors) × 1.30 (if co-medication with CYP3A4 inducers) × 1 (if venous thromboembolism treatment) × 0.849 (if non-valvular atrial fibrillation) × 1.14 (if acute coronary syndromes) × 1.04 (if venous thromboembolism prevention, < 72 h) × 1.29 (if venous thromboembolism prevention, ≥ 72 h)	40.87%			
		V (L)	62.5 × (BW/81) ^0.216^ × (age/61) ^−0.189^ × 0.889 (if female)	19.77%			
		F	0.59 + 0.66 × e^− 0.048 × dose^	/			
Willmann et al. (2018b) [39]	FOCE-I	*k*_a_ (/h)	0.717 (for tablet or diluted suspension) 0.208 (for undiluted suspension)	39.7%	46.6%	Compare PK parameters with that derived from non-compartmental analysis	Comparison of PK parameters with PBPK model predictions
		CL (L/h)	7.26 × (BW/70)^0.323^	26.2%			
		V_c_ (L)	50.9 × (BW/70)	/			
		V_p_ (L)	13.5	/			
		Q (L/h)	0.928	/			
		F	0.648	/			
Zdovc et al. (2019) [32]	Laplacian method with interaction	*k*_a_ (/h)	0.147	794%	59.5%	GOF, VPC, bootstrap	The effect of identified factors on PK
		CL/F (L/h)	6.12 × (ABCB1 expression/1.25)^0.817^	80.8%			
		V/F (L)	96.8	/			
Goto et al. (2020) [18]	NA	*k*_a_ (/h)	0.617 ^d^	58.2% ^d^	13.1 % ^d^	GOF, VPC	NA
		CL (L/h)	5.59 × (CrCl/67.11)^0.159^	41.0% ^d^			
		V (L)	50.9	63.6% ^d^			
		F	1	37.7% ^d^			
Speed et al. (2020) [33]	FOCE-1	*k*_a_ (/h)	0.707	/	46.37%	VPC, bootstrap	The effect of identified factors on PK
		CL/F (L/h)	5.57 × (CrCl_LBM/55)^0.446^	23.02% ^e^			
		V/F	59.4 × (LBM/55)^0.519^	/			
Willmann et al. (2021) [40]	NA	*k*_a_ (/h)	0.799 (for tablet and diluted suspension) 0.226 (for undiluted suspension)	/		NA	Comparison of PK parameters with PBPK model predictions
		CL (L/h)	8.02 × (BW/82.48)^0.481^	27.0%	46.9%		
		V_c_ (L)	53.2 × (BW/82.48)^0.821^	/			
		V_p_ (L)	13.5 × (BW/82.48)^0.821^	/			
		Q (L/h)	2.5 × (BW/82.48)^0.761^	/			
		F	0.59 + 0.66 × e^− dose/BW × 3.97^	25.1%			
Esmaeili et al. (2022) [38]	SAEM	*k*_a_ (/h)	0.821 ^f^	/	38%	GOF, bootstrap	NA
		CL/F (L/h)	3.7 × (CrCl/62.3)^0.89^ × (CTP/5.7)^−1.76^	61%			
		V/F (L)	59	21%			
Liu et al. (2022) [34]	FOCE-I	*k*_a_ (/h)	0.617 ^d^	/	33.6%	GOF, VPC, bootstrap	The effect of identified factors on PK and PD, and dose modification
		CL/F (L/h)	5.03 × (eGFR/80)^0.53^	35.4%			
		V/F (L)	40.3	/			
Singkham et al. (2022) [35]	FOCE-I	*k*_a_ (/h)	0.697	75.91%	0.092 mg/L	GOF, VPC, bootstrap	The effect of identified factors on PK, and dose modification
		CL/F (L/h)	4.19 × (CrCl/57.5)^0.277^	21.94%			
		V/F (L)	37.5 × (BW/63)^0.412^	/			
Willmann et al. (2022) [41]	NA	*k*_a_ (/h)	0.799	/	51.9%	GOF	Comparison of PK parameters with PBPK model predictions, and simulation for dose-exposure relationship
		CL (L/h)	6.07 × (BW/82.48)^0.481^	31.8%			
		V_c_ (L)	53.2 × (BW/82.48)^0.821^	/			
		V_p_ (L)	59.1 × (BW/82.48)^0.821^	/			
		Q (L/h)	2.5 × (BW/82.48)^0.761^	/			
		F	0.752 (≥5 years) 1.2 (<5 years)	40.1%			
Zhang et al. (2022) [37]	FOCE-I	*k*_a_ (/h)	0.821 ^f^	/	36.6% 2.51 μg/L	GOF, VPC, bootstrap	The effect of identified factors on PK, and dose modification
		CL/F (L/h)	5.79 × e^(CRCl − 76.1) × 0.00586 ×^ e^-(TBIL − 14) × 0.0144^ × 1.476 (if *ABCB1* rs4728709 AA/GA)	38.3%			
		V/F (L)	51.5 × e^(BW−66) × 0.00873^	18.5%			
Zhao et al. (2022) [20]	FOCE-I	*k*_a_ (/h)	0.406 × 0.830 (if postprandial status) ^g^	/	21.0% 1.95 μg/L	GOF, bootstrap	The effect of identified factors on PD
		D (h)	0.101 × 4.9 (if postprandial status) ^g^	183% ^g^			
		ALAG (h)	0.164 ^g^	/			
		CL/F (L/h)	7.39 × (CrCl/95)^0.61003^	47.1%			
		V_c_/F (L)	10.9 × (BMI/22.85)^1.364 g^	53.8% ^g^			
		V_p_/F (L)	50.9 ^g^	68.1% ^g^			
		Q/F (L/h)	4.4 ^g^	77.9% ^g^			
		F	0.867 (15 mg, compared to 10 mg) ^g^ 0.608 (20 mg, compared to 10 mg) ^g^ × 1.244 (if postprandial status) ^g^	15.5% ^g^			
Zhang et al. (2023) [36]	FOCE-I	*k*_a_ (/h)	0.821 ^f^	/	20 % 0.193 μg/L	GOF, VPC, bootstrap	The effect of identified factors on PK, and dose modification
		CL/F (L/h)	3.68 × (eGFR/73.275)^0.528^ × (TBIL/13.63)^−0.246^ × 1.257 (if *ABCB1* rs1045642 CT/TT)	/			
		V/F (L)	42.9	/			

Abbreviations: ABCB1: ATP-binding cassette sub-family B member; ALAG: the absorption lag time; ALB: albumin; ALT: alanine aminotransferase; BMI: body mass index; BSA: body surface area; BSV: between-subject variability; BUN: blood urea nitrogen; BW: body weight; CL: clearance; CL/F: apparent clearance; CrCl: creatinine clearance; CrCl_LBM: creatinine clearance calculated using lean body mass; CTP: transformed Child–Turcotte–Pugh Score; CYP3A4: cytochrome P450 3A4; D: duration; DVT: patients with deep vein thrombosis; F: bioavailability; FOCE: first-order conditional estimation; FOCE-I: first-order conditional estimation with interaction; GOF: goodness-of-fit; HCT: hematocrit; HRS: patients with hip replacement surgery; INH: CYP3A4 inhibitors or P-gp inhibitors; IOV: inter-occasion variability; *k*_a_, absorption rate; KRS: patients with knee replacement surgery; LBM: lean body mass; OFV: objective function value; PBPK: physiologically based pharmacokinetic; pc-VPC: prediction-corrected visual predictive check; PD: pharmacodynamics; PE: prediction error; P-gp: P-glycoprotein; PK: pharmacokinetics; Q: inter-compartment clearance; Q/F: apparent inter-compartment clearance; SAEM: stochastic approximation expectation-maximization; SCr: serum creatinine; TBIL: total bilirubin; V: volume of distribution; V_c_: central volume of distribution; V_c_/F: apparent central volume of distribution; V/F: apparent volume of distribution; V_p_: peripheral volume of distribution; VPC: visual predictive check; V_p_/F: apparent peripheral volume of distribution. ^a^ The typical value of CL for patients with KRS after surgery < 3 days was not reported. ^b^ The BSV of CL for patients with KRS was not reported. ^c^ Correlation between CL and V was 0.729. ^d^ The estimates were fixed to values reported by Kaneko et al. [25]. ^e^ The BSV of CL was Box-cox transformed with an estimate of lambda −1.83. ^f^ The estimates were fixed to values reported by Willmann et al. [31]. ^g^ The estimates in patients were fixed to values estimated according to the study conducted in healthy subjects by Zhao et al. [20].

(6) $$M=M_{0}\times \left(1-\frac{E_{max}\times C_{p}}{EC_{50}+C_{p}}\right)$$

(7) $$M=M_{0}\times \left(1-\frac{E_{max}\times C_{p}^{Hill}}{EC_{50}^{Hill}+C_{p}^{Hill}}\right)$$

(8) $$M=\frac{E_{max}\times C_{p}^{Hill}}{EC_{50}^{Hill}+C_{p}^{Hill}}$$

where *M* represents the measurement of FXa activity (aPTT, or anti-Xa), *M*_0_ represents the baseline FXa activity (aPTT, or anti-Xa), *E_max_* represents the maximum FXa activity (aPTT, or anti-Xa), *EC*_50_ represents the rivaroxaban concentration generating 50% of the maximum FXa activity (aPTT, or anti-Xa), *Hill* represents the exponent of rivaroxaban concentration, and *C_p_* represents the plasma concentration of rivaroxaban.

Anti-Xa activity was correlated with rivaroxaban concentration in a near-linear (Equation (5)) or E_max_ model (Equation (8)) [20,38]. aPTT was expressed as a linear (Equation (2)), near-linear (Equation (4)), or E_max_-related relationship (Equation (6)) [19,26,32,38]. Heptest was expressed as an E_max_-related relationship (Equations (6) and (7)) [19,27] and PiCT showed a near-linear relationship (Equation (4)) [27]. The final PD parameters of the included studies are listed in Table 3 and Table S2.

#### 3.3.4. Model Evaluation

All models were internally evaluated using goodness-of-fit plots, non-parametric bootstrapping, visual predictive check (VPC), and prediction-corrected VPC (pc-VPC). Three studies conducted an internal evaluation by comparing PK parameter estimates with physiologically based pharmacokinetic (PBPK) model predictions [39,40,41].

#### 3.3.5. Model Application

Eleven studies performed Monte Carlo simulations to explore the effects of the identified covariates on PK or PD parameters [20,23,24,27,31,32,33,34,35,36,37]. Four studies proposed further recommendations for dose regimens in different subgroups [27,34,36,37]. 

### 3.4. Comparison of Studies

The simulated concentration-time and PD biomarker-time profiles at the steady state of the included studies were compared. Detailed information on the simulated patient characteristics is provided in Table S3. For a comparison of PK parameters, apparent clearance (CL/F) was chosen because it is the most important PK parameter for long-term pharmacotherapy and could be compared directly among studies that employed different dosing regimens [42]. As for the PD index, FXa activity was selected for comparison because it directly reflects the inhibitive effect of rivaroxaban. PT, which was investigated in most studies, was also compared. Other PD biomarkers were not assessed because of a lack of adequate data or evidence of their clinical relevance.

Regarding the PK comparison, studies with overlapping data and smaller sample sizes were excluded [21,22,23,24,27,28,29]. Therefore, 17 studies were used to compare the PK parameters [18,19,20,25,26,30,31,32,33,34,35,36,37,38,39,40,41], and 13 studies were used for PD [19,20,21,22,23,24,25,26,27,30,32,34,38] analyses. Studies conducted on patients who were predominantly Caucasians were grouped into Caucasian models.

#### 3.4.1. Pharmacokinetic Analysis

The dose-dependent bioavailability was reported in nine studies [19,20,21,22,23,24,31,40,41]. For studies conducted on patients with NVAF, bioavailability was estimated in a dose-dependent manner only by Willmann et al. [31], but it was fixed or not estimated in other studies. Therefore, the CL/F values in different dose groups were compared.

**Table 3:** Final population pharmacodynamic parameters of included studies.

Study (Year)	Analytical Method/ Regent	Formula	Fixed Effect Parameters		Between-Subject Variability	Residual Unexplained Variability
**Prothrombin time**						
Mueck et al. (2007) [19]	Neoplastin Plus^®^	PT = PT_0_ + slope × C_p_	PT_0_ (s)	12.7	3.7%	1.58 s
			slope (s/(μg/L))	0.0458	12.3%	
Mueck et al. (2008b) [22] ^a^	STA^®^ Neoplastine^®^	PT = PT_0_ + slope × C_p_	PT_0_ (s)	13.1 × [1 + 0.034 × (ALB − 3.3)] × [1–0.0045 × (CrCl − 96)] (HRS) 13.4 × [1 + 0.05 × (ALB − 3.6)] × [1–0.008 × (CrCl − 104)] (KRS)	5.8% (HRS) 7.4% (KRS)	8.6% (HRS) 10.9% (KRS)
			slope (s/(μg/L))	0.032 (HRS) 0.042 (KRS)	43.2%	
Mueck et al. (2011) [23]	STA^®^ Neoplastine^®^	PT = PT_0_ + slope × C_p_^1-Hill × Cp^	PT_0_ (s)	12.5 × [1–0.0004 × (CrCl − 87.4)]	9.7%	10.3%
			slope (s/(μg/L))	0.036	/	
			Hill	0.0000996 × [1 + 0.0046 × (CrCl − 87.4)]	4.3%	
Xu et al. (2012) [24]	STA Neoplastin CI Plus^®^	PT = PT_0_ + slope × C_p_^1-Hill × Cp^	PT_0_ (s)	13.9 × [1–0.0003 × (CrCl − 96.9)]	9.32%	7.6%
			slope (s/(μg/L))	0.032	/	
			Hill	0.0000593 × [1 + 0.0233 × (CrCl − 96.9)]	6.61%	
Kaneko et al. (2013) [25]	Neoplastin Plus^®^	PT = PT_0_ + slope × C_p_^1-Hill × Cp^	PT_0_ (s)	11.4 × [1 + 0.0035 × (age − 70.98) + 0.00242 × (LBM − 49.69) − 0.065 × (ALB − 4.22) − 0.015 × (HGB − 14.06)]	9.6%	7.1%
			slope (s/(μg/L))	0.0467	/	
			Hill	0.000155 × (TBIL/14.02)^−1.11^	7.3%	
Tanigawa et al. (2013) [26]	Neoplastin Plus^®^	PT = PT_0_ + slope × C_p_^Hill^	PT_0_ (s)	13.7	8.0%	9.4%
			slope (s/(μg/L))	0.0227	27.0%	
			Hill	1.1	/	
Girgis et al. (2014) [27]	STA Neoplastin CI Plus^®^	PT = PT_0_ + slope × C_p_^1-Hill × Cp^	PT_0_ (s)	11.4 × [1–0.000192 × (CrCl − 76)]	22.6%	12.9%
			slope (s/(μg/L))	0.0426	4.42%	
			Hill	0.0000551 × [1 + 0.0174 × (CrCl − 76)]	/	
Suzuki et al. (2018) [30]	Neoplastin Plus^®^	PT = PT_0_ + slope × C_p_	PT_0_ (s)	14	/	0.87 s
			slope (s/(μg/L))	0.0335 × (HCT/42)^−1.10^	11.7%	
	Thromborel^®^ S	PT = PT_0_ + slope × C_p_	PT_0_ (s)	12.5	/	0.71 s
			slope (s/(μg/L))	0.0158 × (HCT/42)^−1.38^ × (ALB/4.2)^−2.31^	8.7%	
	ThromboCheck PT^®^	PT = PT_0_ + slope × C_p_	PT_0_ (s)	11.8	/	0.61 s
			slope (s/(μg/L))	0.0220 × (HCT/42)^−1.30^	9.5%	
	RecombiPlasTin 2G^®^	PT = PT_0_ + slope × C_p_	PT_0_ (s)	12.1	/	0.75 s
			slope (s/(μg/L))	0.0323	11.2%	
Suzuki et al. (2018) [30]	Thrombocheck PT Plus^®^	PT = PT_0_ + slope × C_p_	PT_0_ (s)	13.2	/	0.82 s
			slope (s/(μg/L))	0.0266 × (HCT/42)^−1.07^ × (age/69)^0.680^	10.6%	
Zdovc et al. (2019) [32]	Thromborel^®^ S	PT = PT_0_ + slope × log(C_p_)	PT_0_ (s)	12.8	11.4%	1.85 s
			slope (s/(μg/L))	0.215	109%	
Liu et al. (2022) [34]	Thromborel^®^ S	PT = PT_0_ + slope × C_p_	PT_0_ (s)	13.9 × e^0.00574 × (TBIL − 12)^ × (1 − 0.0872 × eGFR/80)	5.6 %	9.4%
			slope (s/(μg/L))	0.0133	61.8 %	
Esmaeili et al. (2022) [38]	Fisherbrand^TM^	PT = PT_0_ + slope × C_p_	PT_0_ (s)	12.6	2%	12%
			slope (s/(μg/L))	0.018	54%	
Zhao et al. (2022) [20]	Thromborel^®^ S	PT = PT_0_ + slope × C_p_^Hill^	PT_0_ (s)	11.4 × (BW/68.2)^−0.159^ × (TCHO/3.96)^−0.0794 b^	6.9% ^b^	0.372 s
			slope (s/(μg/L))	0.0018 ^b^	24.9% ^b^	
			Hill	1.37 ^b^	/	
**Factor Xa activity**						
Mueck et al. (2007) [19]	Two-step photometric assay	FXa = FXa_0_ × (1 − $\frac{E_{\max}{\;\times \;C}_{p}}{{\mathrm{EC}}_{50}\;+\;\mathrm{Cp}}$)	FXa_0_ (U/mL)	0.87	10.09%	0.0027 U/mL
			E_max_ (U/mL)	0.86	9.9%	
			EC_50_ (μg/L)	220	/	
Mueck et al. (2008b) [22]	Two-step photometric assays	FXa = FXa_0_ × (1 − $\frac{E_{\max}{\;\times \;C}_{p}}{{\mathrm{EC}}_{50}\;+\;\mathrm{Cp}}$)	FXa_0_ (U/mL)	1	13.9% (HRS) 14.5% (KRS)	8.4% (HRS) 9.7% (KRS)
			E_max_ (U/mL)	0.881 (day 1, HRS) 0.942 (steady state, HRS) 0.837 (KRS)	/	
			EC_50_ (μg/L)	296 (HRS) 243(day 1, KRS) 172 (steady state, KRS)	36.6% (HRS) 50.2% (KRS)	
Tanigawa et al. (2013) [26]	Two-step photometric assays	FXa = FXa_0_ × $(1-\frac{E_{\max}{\;\times \;C}_{p}{}^{\mathrm{hill}}}{{\mathrm{EC}}_{50}{}^{\mathrm{hill}}{\;+\;C}_{p}{}^{\mathrm{hill}}})$	FXa_0_ (U/mL)	0.803 × [1 − 0.00656 × (age − 65.59)]	4.8%	6.9%
			E_max_ (U/mL)	0.928	/	
			EC_50_ (μg/L)	221	10.6%	
			Hill	1.16	/	
Girgis et al. (2014) [27]	Two-step photometric assays	FXa = FXa_0_ × $\left(1-\frac{E_{\max}{\;\times \;C}_{p}}{{\mathrm{EC}}_{50}\;+\;\mathrm{Cp}}\right)$	FXa_0_	104% ^c^	16.61%	10.05%
			E_max_	107% ^c^	/	
			EC_50_ (μg/L)	760	5.97%	

Abbreviations: ALB: albumin; BW: body weight; C_p_: rivaroxaban plasma concentration; CrCl: creatinine clearance; EC_50_: concentration generating 50% of the maximum effect; eGFR: estimated glomerular filtration rate; E_max_: the maximum effect; FXa: factor Xa; FXa_0_: baseline of FXa; HCT: hematocrit; HGB: hemoglobin; HRS: patients with hip replacement surgery; KRS: patients with knee replacement surgery; LBM: lean body mass; PT: prothrombin time; PT_0_: baseline of PT; TBIL: total bilirubin; TCHO: total cholesterol. ^a^ Complex PT model with components describing recovery of clotting factors to pre-surgery levels over time was developed by Mueck et al. (2008b) [22], and the simplified formula was provided in the table. ^b^ The estimates in patients with NVAF were fixed to values estimated from healthy subjects by Zhao et al. [20]. ^c^ Data were expressed as a percentage of factor Xa activity in control plasma. Notes: Study by Mueck et al. (2008a) [21] also applied PT to develop PK/PD model, but did not report the parameter estimates.

**Figure 1:**
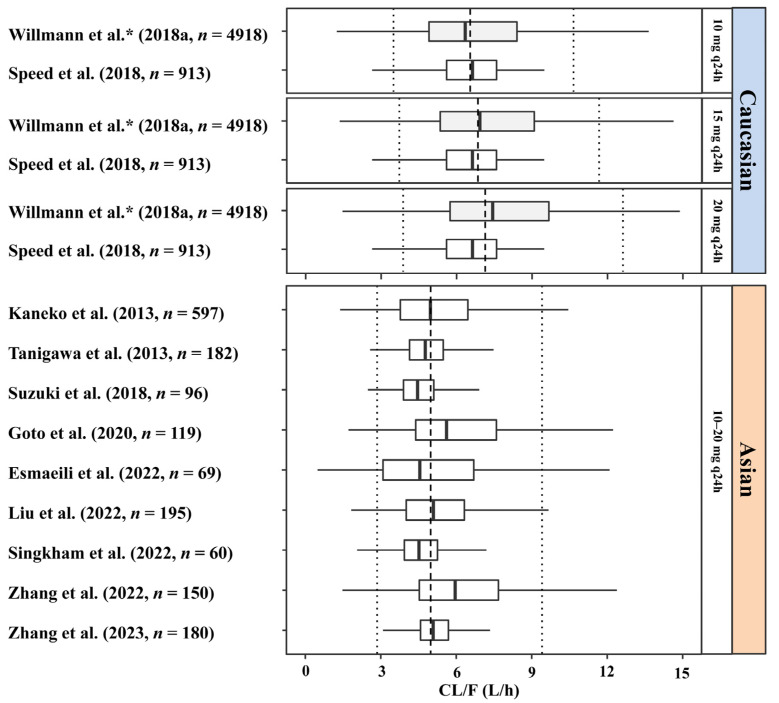
Apparent clearance of rivaroxaban in adult Caucasian and Asian patients with non-valvular atrial fibrillation taking 10–20 mg rivaroxaban. The typical patients of Caucasian and Asians were set as male, 60 years old, and had the following demographic parameters: body weight, 70 kg; lean body mass (LBM), 50 kg; serum creatinine, 1 mg/dL; creatinine clearance (CrCl, or estimated glomerular filtration rate [eGFR]), 80 mL/min; CrCl calculated with LBM, 60 mL/min; blood urea nitrogen, 16 mg/dL; hematocrit, 40%; albumin, 4 g/dL; alanine aminotransferase, 22 IU/L; total bilirubin, 14 μmol/L; transformed Child–Turcotte–Pugh Score, 5.7; *ABCB1* expression, 1.25; *ABCB1* rs1045642, TT; *ABCB1* rs4728709, GG; and without the co-administration of CYP3A4 inducers/inhibitors or P-gp inhibitors. The dashed and dotted lines in each panel represent the median, 5th, and 95th percentiles of the apparent clearance in each corresponding population, respectively. * The dose-dependent bioavailability (F) was estimated in study by Willmann et al. [18,25,26,30,31,33,34,35,36,37,38].

For patients with NVAF and normal renal function, the CL/F of rivaroxaban in Caucasian adult patients (6.45–7.64 L/h) was 31–43% higher than that in Asians (4.46–5.98 L/h) taking 10–20 mg q24h (Figure 1). However, there was also no obvious difference in the median CL/F among Asians, including Japanese, Chinese, Thai, and Iranian patients with NVAF (Figure S2). The simulated concentration-time profiles at steady state of all included studies were shown in Figure S3.

**Figure 2:**
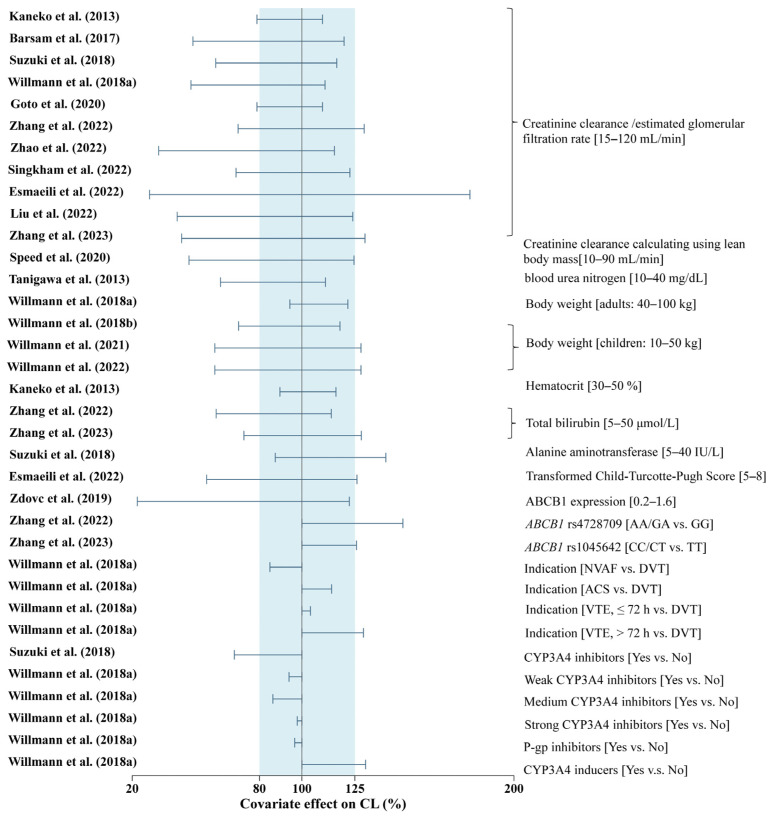
Effect of covariates on the apparent clearance of rivaroxaban. The horizontal bars represent the effect of each covariate on clearance (CL) in each study. The effect of each covariate on CL was characterized as the ratio of CL in the range of each covariate to the typical CL. The gray shadow represents the range of 80–125% [18,20,25,26,28,30,31,33,34,35,36,37,38,39,40,41].

Covariates on the CL of rivaroxaban in previous studies included renal function [CrCl, eGFR, and blood urea nitrogen (BUN)], hepatic function [alanine aminotransferase (ALT), total bilirubin (TBIL), transformed Child–Turcotte–Pugh Score (CTP)], genetic polymorphisms of *ABCB1*, and the presence of co-medication. The effects of the covariates on CL in each study are shown in Figure 2.

Eleven studies identified renal function as significant covariates on the CL of rivaroxaban, including CrCl, eGFR, or BUN. The impact of CrCl on CL varied in these studies. Compared with patients with CrCl (or eGFR) of 80 mL/min, moderately (CrCl (or eGFR) 30–49 mL/min) or severely impaired renal function (CrCl (or eGFR) 15–29 mL/min) may lead to a decrease in the CL of approximately 4–50% and 12–72%, respectively. The change in CL/F per unit renal function was similar between Asian and Caucasian patients but had a large variability. For example, the CL/F of rivaroxaban decreased by 0.13–5.58% in the Asian population and 0.34–2.84% in the Caucasian population per 1 mL/min decrease in CrCl (or eGFR) from 15 to 120 mL/min (Figure S4).

A significant effect of hepatic function on CL was reported in four studies, which were characterized by the ALT, TBIL, and CTP levels [30,36,37,38]. Increased TBIL (>35 μmol/L) or CTP (>6.5) levels may lead to a decrease in the CL/F of rivaroxaban by >20% (Figure 2). Nevertheless, the identified liver indices were different in each of these studies, which warrants further evaluation.

Body weight (BW) was also identified as a significant covariate in three studies conducted on pediatric patients [39,40,41] (Figure 2). The body-weight-adjusted CL/F in children was higher than that in adults. For Caucasian children weighing 10, 30, and 50 kg, the median body weight-adjusted CL/F was reported to be approximately 2.05-, 1.47-, and 1.16-fold higher than that in adults, respectively (Figure S5). BW was also assessed in 15 studies conducted on adult patients [20,21,22,23,25,28,29,30,31,32,33,34,35,36,37,38], but only one study by Willmann et al. [31] found an increase in CL with a decrease in the BW of patients, which may not have clinical relevance (Figure 2).

The impact of genetic polymorphisms of *ABCB1* (rs1045642, rs4148738, or rs4728709) on CL was reported, with an effect ranging from 19 to 57% among patients [34,36,37]. The study conducted by Zdovc et al. [32] identified the relative expression of the *ABCB1* gene determined using the comparative Ct method as a significant covariate for CL in 17 patients [32].

Furthermore, co-medication, including CYP3A4 inhibitors, inducers, and P-gp inhibitors, was found to notably affect CL in two studies [30,31]. Co-medication with CYP3A4 inducers increased CL by approximately 30% compared to rivaroxaban monotherapy [31]. The influence of strong, moderate, and weak CYP3A4 inhibitors and P-gp inhibitors on CL was < 15% in a study by Willmann et al. [31]. In contrast, it was approximately 32% in the study by Suzuki et al. [30].

#### 3.4.2. Pharmacodynamics Analysis: PT

Most studies displayed similar VPDs in the PT-time profiles (Figure S6), except for the study by Zdovc et al. [32], which may be due to the imprecise estimation of the PK and PD parameters in that study (ω_ka_: 794%; ω_CL_: 81%; ω_slope_: 109%). Therefore, that study was excluded from further analyses.

**Figure 3:**
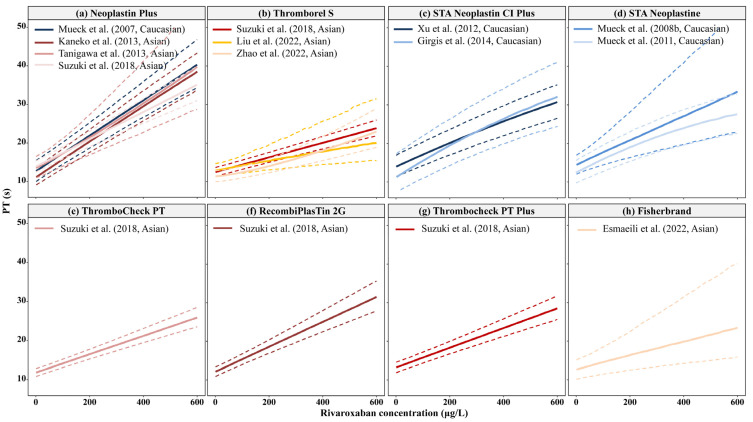
Relationship between prothrombin time (PT) and rivaroxaban concentration between Asian and Caucasian populations using different PT thromboplastin reagents. The reagents used in the included studies were the following: (**a**) Neoplastion Plus, (**b**) Thromborel S, (**c**) STA Neoplastin CI Plus, (**d**) STA Neoplastine, (**e**) ThromboCheck PT, (**f**) RecombiPlasTin 2G, (**g**) Thrombocheck PT Plus, and (**h**) Fisherbrand. The bold, solid, and dashed lines represent the median, 5th, and 95th percentiles of the simulated PT-rivaroxaban concentration profiles in each model, respectively. Zdovc et al. reported PT models with a reagent named Thromborel S. Because the PT-time profile of that model was significantly different from the others (Supplementary Figure S3), it was excluded from the subgroup analysis [19,20,22,23,24,25,26,27,30,34,38].

The relationship between rivaroxaban concentration and PT according to the type of PT reagent used is shown in Figure 3. As illustrated in Figure 3a (Neoplastin Plus), no obvious difference between Asian and Caucasian populations was observed when the same bioassay for PT was used. Moreover, the estimated baseline PT (PT_0_) was similar between Asian (11.4–14 s) and Caucasian (11.4–13.9 s) populations, as listed in Table 3. The estimated change in PT per unit rivaroxaban concentration change (slope) was also similar between Asians [0.0018–0.0467 s/(μg/L)] [20,25,26,30,34,38] and Caucasians [0.032–0.0458 s/(μg/L)] [19,22,23,24,27,32], but with large variability, which may be due to the different PT reagents employed in these studies.

Renal function (CrCl, eGFR), hepatic function [albumin (ALB), TBIL], demographics (age, BW), hemoglobin (HGB), and total cholesterol were identified as significant covariates for PT_0_ [20,22,23,24,25,27,34]. However, the impact of these covariates was limited compared to the PT change due to increased rivaroxaban concentration. HCT, ALB, and age were identified as significant covariates on the slope in the study by Suzuki et al. [30], which may deserve further evaluation (Figure S7).

#### 3.4.3. Pharmacodynamics Analysis: FXa Activity

**Figure 4:**
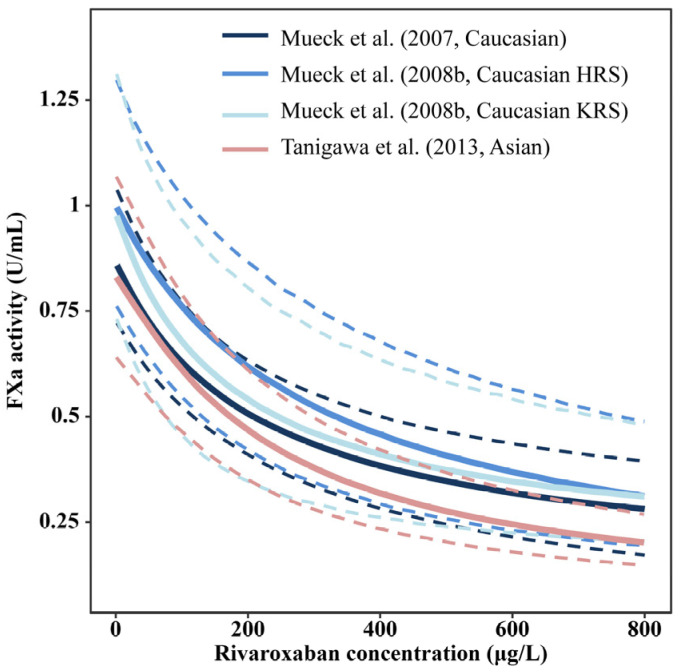
Relationship between Factor Xa activity and rivaroxaban concentration between Asian and Caucasian. The bold, solid, and dashed lines represent the median, 5th, and 95th percentiles of the simulated Factor Xa activity-rivaroxaban concentration profiles in each study, respectively. HRS: patients with hip replacement surgery; KRS: patients with knee replacement surgery [19,22,26].

The relationship between FXa activity and rivaroxaban concentration in Asians was similar to that in Caucasians (Figure 4). The estimates of parameters related to FXa activity in the Asian population (FXa_0_: 0.803 U/mL; E_max_: 0.928 U/mL; EC_50_: 221 μg/L) [26] were comparable to those in Caucasian populations (FXa_0_: 0.87–1.0 U/mL; E_max_: 0.86–0.942 U/mL; EC_50_: 172–296 μg/L) using the same bioassay for FXa activity determination as listed in Table 3.

The VPDs of FXa activity-time profiles at steady state in all included studies are shown in Figure S8. Few covariates have been reported for FXa activity. Only the study by Girgis et al. [27] found that age had an impact on baseline FXa activity, but no details were provided. Because only one study was conducted on Japanese patients and two studies were conducted in a non-Japanese population, further investigation may need to be performed.

## 4. Discussion

Rivaroxaban is a DOAC that was first approved in 2008 and is currently widely used globally, for the treatment and prevention of thromboembolic disorders. However, whether a lower rivaroxaban dose is required for Asian people is still debatable. To our knowledge, this is the first systematic review to summarize the knowledge regarding such potential ethnic differences from the perspective of the population PK and PK/PD profiles of rivaroxaban. 

Population PK studies conducted in real-world patients showed that the median CL/F of rivaroxaban in adult Caucasian patients was approximately 31–43% higher than in Asian patients with normal renal and hepatic function. This finding is also supported by phase II and III clinical studies conducted on Japanese patients with NVAF [25,26]. An approximate one-third decrease in the CL/F in Asians might lead to the requirement of one-third reduction in the rivaroxaban dose, as compared to that for Caucasians. The effects of BW, age, renal function, and hepatic function could not explain the difference. 

The difference in CL/F may be partially attributed to the genetic polymorphism of *ABCB1*. A population PK study conducted by Zhang et al. [37] identified that patients carrying *ABCB1* (rs 4728709) AA/GA had a 47.6% higher CL/F than those with GG. Moreover, Zhang et al. [36] identified that patients carrying *ABCB1* (rs1045642) CT/TT had a 25.7% higher CL/F than those with a CC polymorphism [36]. The frequency of *ABCB1* (rs 4728709) AA/GA in American and African-American populations was determined to be approximately 2.1-fold higher than that in Asians. Moreover, it was also reported that the trough rivaroxaban concentration between different genotypes of *ABCB1* at the rs128503 locus was significantly different [43]. However, all of these studies were performed in a Chinese population, and further studies in other ethnicities may be considered to explore the impact of genetic polymorphisms.

Moreover, food intake may also play a role in the differences in CL/F because the bioavailability of rivaroxaban is food-dependent upon administration at doses between 15 and 20 mg. Differences in dietary habits and diet content among ethnicities may also explain the differences observed. However, information about food, such as meal timing and dietary content, was not recorded in most of the studies and may deserve further investigation.

According to European Medicines Agency, no clinically relevant inter-ethnic differences are observed among Caucasian, African-American, Hispanic, Japanese, or Chinese patients regarding rivaroxaban PK and PD characteristics [44]. However, the statement was different from the Food and Drug Administration (FDA), which reported that healthy Japanese subjects have 20–40% higher exposures on average, compared to those in other ethnicities including Chinese, and these differences in exposure are reduced when corrected for body weight [45]. The reason for this different statement is unclear. 

The PK and PD characteristics in Chinese healthy subjects (*n* = 8) taking a single dose was reported to be in line with that in Caucasian subjects when not corrected by body weight, even though a 47% lower area under the concentration–time curve (AUC) per body weight was observed in Chinese individuals [46]. However, AUC at steady state in Caucasian healthy subjects taking multiple doses of rivaroxaban were 1.32- to 1.51-fold of that in Chinese subjects [46,47]. Data from healthy subjects might not fully represent patients in the real world. Population analyses of real-world data could thus contribute to comprehensively delineating PK and PD characteristics.

Previous exposure–response analyses based on patients with NVAF showed that the risk of ischemic stroke, non-central nervous system systematic embolism, or all-cause death in Asians was not significantly different from that in individuals from other regions. However, Asians had a statistically significantly higher risk of major or non-major clinically relevant bleeding compared to that in West European and Latin American individuals [48]. The different risks of bleeding between geographic regions were also reported in other types of patients [49,50]. This could be partially explained by the other confounding factors, such as history of gastrointestinal bleeding, baseline use of non-steroidal anti-inflammatory drugs or aspirin, and age [48]. Moreover, the higher risk of bleeding in Asians might be attributed to higher rivaroxaban exposure compared to that in other ethnicities with the same dose regimen.

Renal function has a significant impact on the CL/F of rivaroxaban because 1/3 of the drug is eliminated unchanged in the urine, and about half of the metabolites are eliminated by the kidney. The change in CL/F by unit renal function has large variability among studies, ranging from 0.13 to 5.58%, but no obvious difference was observed between Asian and Caucasian populations.

Although approximately 2/3 of rivaroxaban is metabolized by hepatic enzymes [4], only 4 of 24 studies have reported the effect of the hepatic function index on the CL/F of rivaroxaban. The identified indices were ALT, TBIL, and CTP; none were well-recognized in previous studies [30,36,37,38]. This may be because none of these covariates could fully reflect hepatic function.

The effect of age on CL/F was ≤30% and may not have significant clinical relevance as the drug label recommended by the FDA [45]. However, it should be noted that elderly patients in the real world have more concomitant diseases and are prone to have an unstable morbid state [51]. Besides the risk of increased PK exposure, elderly patients also have a higher risk of thrombotic and bleeding events. Therefore, further studies may need to be performed to assess the effects of rivaroxaban treatment on the elderly population.

It has been reported that Caucasian children weighing ≤ 30 kg had at least 47% higher CL/F per body weight (kg) than Caucasian adults. This can be explained by the fact that the ratio of liver to total body mass is larger in children than in adults, resulting in increased blood flow. Stronger hepatic metabolic enzyme activity may also partially contribute to this [52]. Owing to the lack of population PK studies in Asian children, further studies need to be conducted.

The exposure-PT relationship was described as linear or near-linear in Asian and Caucasian patients. The baseline PT and change in PT induced by rivaroxaban concentration in Asians were not significantly different from that in Caucasian populations, suggesting no distinct difference between Asian and Caucasian populations. However, it should be noted that PT may not be a specific biomarker for rivaroxaban and could be influenced by the bioassay method and test reagents used.

In addition, the relationship between exposure and FXa activity in the Asian population was similar to that in the Caucasian population, as described by the E_max_ model, revealing comparable estimates in baseline FXa, EC_50_, and E_max_. However, only three studies (one in Japanese patients and two in non-Japanese patients) were conducted, which may require further investigation. Moreover, further studies are needed to explore the relationship between FXa activity and clinical outcomes, such as the rate of bleeding and thrombotic events.

Considering the lower CL/F of rivaroxaban in Asians than in Caucasians and the similar PK/PD relationship among ethnicities, it could be inferred that a lower dose of rivaroxaban is required for Asians. This finding is supported by studies based on other Asian populations, except for Japanese individuals [10,11,12]. Meanwhile, the efficacy of a lower rivaroxaban dose was also confirmed with Japanese patients in real world clinical settings [53]. However, owing to inconsistent findings [54,55,56], further studies with larger sample sizes are warranted for verification.

Our study had several limitations. First, because only literature published in English was included in our analysis, studies published in other languages were omitted. However, this may not essentially impact our conclusion because studies published in other languages may be limited by their small sample size. Second, we did not assign weights to studies with different sample sizes and reporting quality when comparing PK/PD characteristics. However, approximately 3/4 of the included studies had a sample size greater than 100 and a compliance rate ≥ 80%, which means that they should be recognized as well-reported. 

## 5. Conclusions

In this review, the potential ethnic difference in the PK/PD of rivaroxaban was evaluated from the perspective of a population analysis. Approximately 31–43% lower CL/F of rivaroxaban was observed in Asians than Caucasians. However, the relationship between rivaroxaban concentration and PT or FXa activity was similar between the two ethnicities. Renal function was identified as a significant covariate of the CL/F of rivaroxaban, and no well-recognized covariates significantly affected PT or FXa activity. A lower dose of rivaroxaban might be required for Asians, and further studies are needed to explain the difference in the CL/F of rivaroxaban between Asian and Caucasian populations, which is essential for optimal patient dose regimens.

## Data Availability

All data generated or analyzed during this study are included in this article.

## Supplementary Materials

The following supporting information can be downloaded at: https://www.mdpi.com/article/10.3390/pharmaceutics15020588/s1, Figure S1: PRISMA flow diagram for identifying population pharmacokinetic and population pharmacokinetic-pharmacodynamic studies of rivaroxaban; Figure S2: Concentration-time profiles of rivaroxaban at steady state; Figure S3: Distribution of apparent clearance of rivaroxaban in Japanese patients, Chinese patients, and other Asian patients with non-valvular atrial fibrillation; Figure S4: The apparent clearance of rivaroxaban versus renal function as reported in population pharmacokinetic models; Figure S5: The apparent clearance per body weight in pediatric and adult patients; Figure S6: Prothrombin time-time profiles of rivaroxaban at steady state for (a) Asian patients with NVAF and (b) Caucasian patients with or without NVAF; Figure S7: Effect of covariates on baseline PT (a) and slope (b); Figure S8: FXa activity-time profiles of rivaroxaban at steady state; Table S1: Checklist for literature quality when reporting a clinical pharmacokinetic study; Table S2: Final population pharmacodynamic parameters of the included studies; Table S3: Demographic information of the simulated patients.
