# Supplementary material for: Is a Lower Dose of Rivaroxaban Required for Asians? A Systematic Review of a Population Pharmacokinetics and Pharmacodynamics Analysis of Rivaroxaban
Source: Pharmaceutics. 2023 Feb 9;15(2):588. doi: 10.3390/pharmaceutics15020588 (PMC9964148; doi:10.3390/pharmaceutics15020588)
Supplement: Supplementary file 1 [file pharmaceutics-15-00588-s001.zip › supplementary Figures.pdf]

Is a Lower Dose of Rivaroxaban Required for Asians? A Systematic Review of a Population Pharmacokinetics and Pharmacodynamics Analysis of Rivaroxaban

Xiao-Qin Liu [1], Zi-Ran Li [1,2], Chen-Yu Wang [1], Yue-Ting Chen [1] and Zheng Jiao [1,*]

[1] Department of Pharmacy, Shanghai Chest Hospital, Shanghai Jiao Tong University, Shanghai 200030, China
[2] Department of Pharmacy, Huashan Hospital, Fudan University, Shanghai 200437, China
* Correspondence: jiaozhen@online.sh.cn

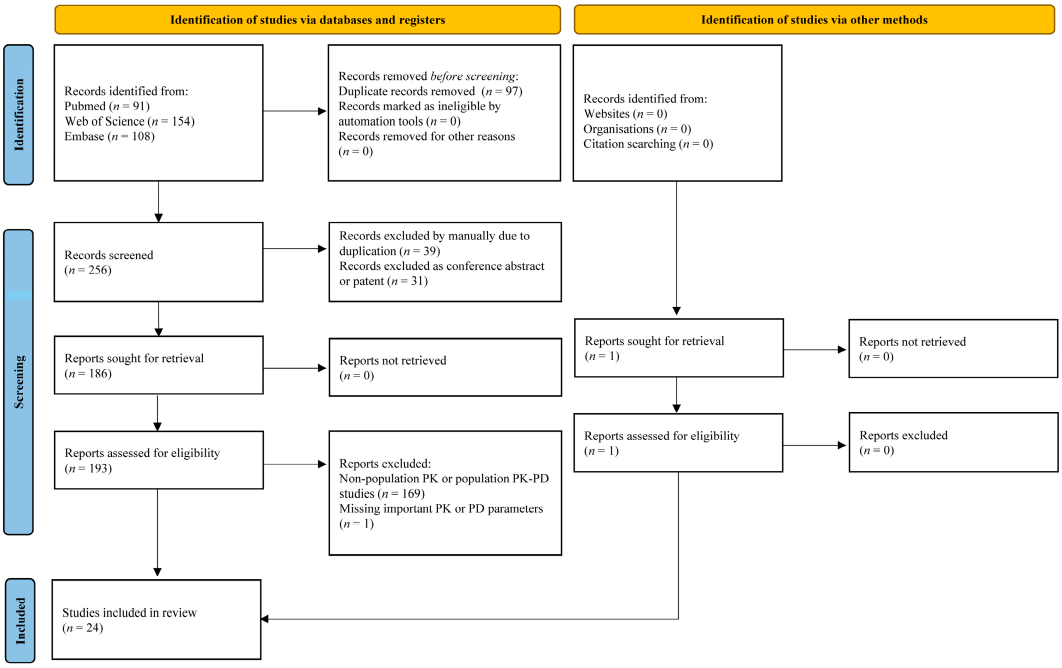

Figure S1. PRISMA flow diagram for identifying population pharmacokinetic and population pharmacokinetic-pharmacodynamic studies of rivaroxaban.

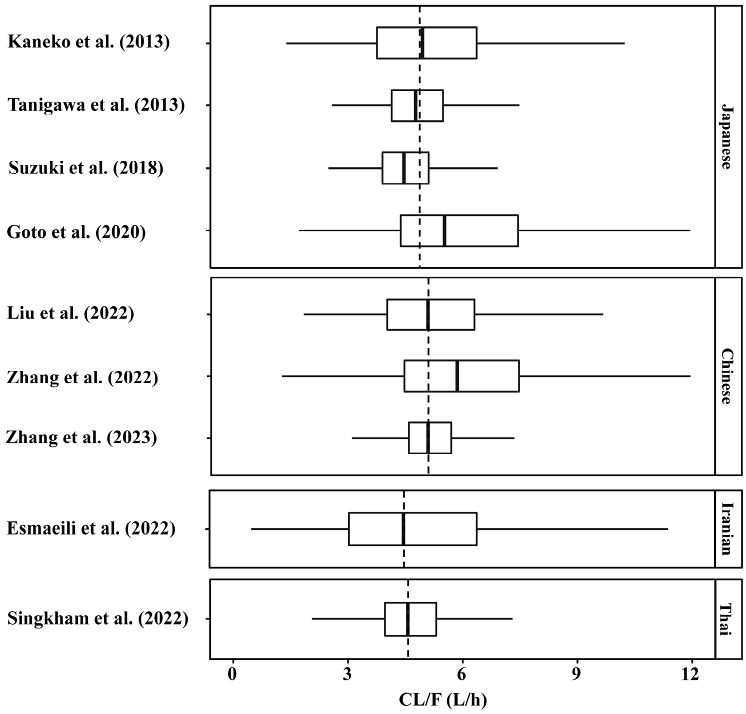

Figure S2. Distribution of apparent clearance of rivaroxaban in Japanese patients, Chinese patients, and other Asian patients with non-valvular atrial fibrillation.

The typical patients were male and had the following parameters: age, 60 years; body weight, 70 kg; serum creatinine, 1 mg/dL; lean body mass (LBM), 50 kg; creatinine, clearance (CrCl, or estimated glomerular filtration rate [eGFR]), 80 mL/min; CrCl calculated using LBM, 60 mL/min; blood urea nitrogen, 16 mg/dL; hematocrit, 40%; albumin, 4 g/dL; alanine aminotransferase, 22 IU/L; total bilirubin, 14 µmol/L; transformed Child-Turcotte-Pugh Score, 5.7; *ABCB1* expression, 1.25; *ABCB1* rs1045642, TT; *ABCB1* rs4728709, GG; and without the co-administration of CYP3A4 inducers/inhibitors or P-gp inhibitors. All patients were assumed to consume 20 mg of rivaroxaban qd with food. The dashed lines in each panel represent the median CL/F in the corresponding population.

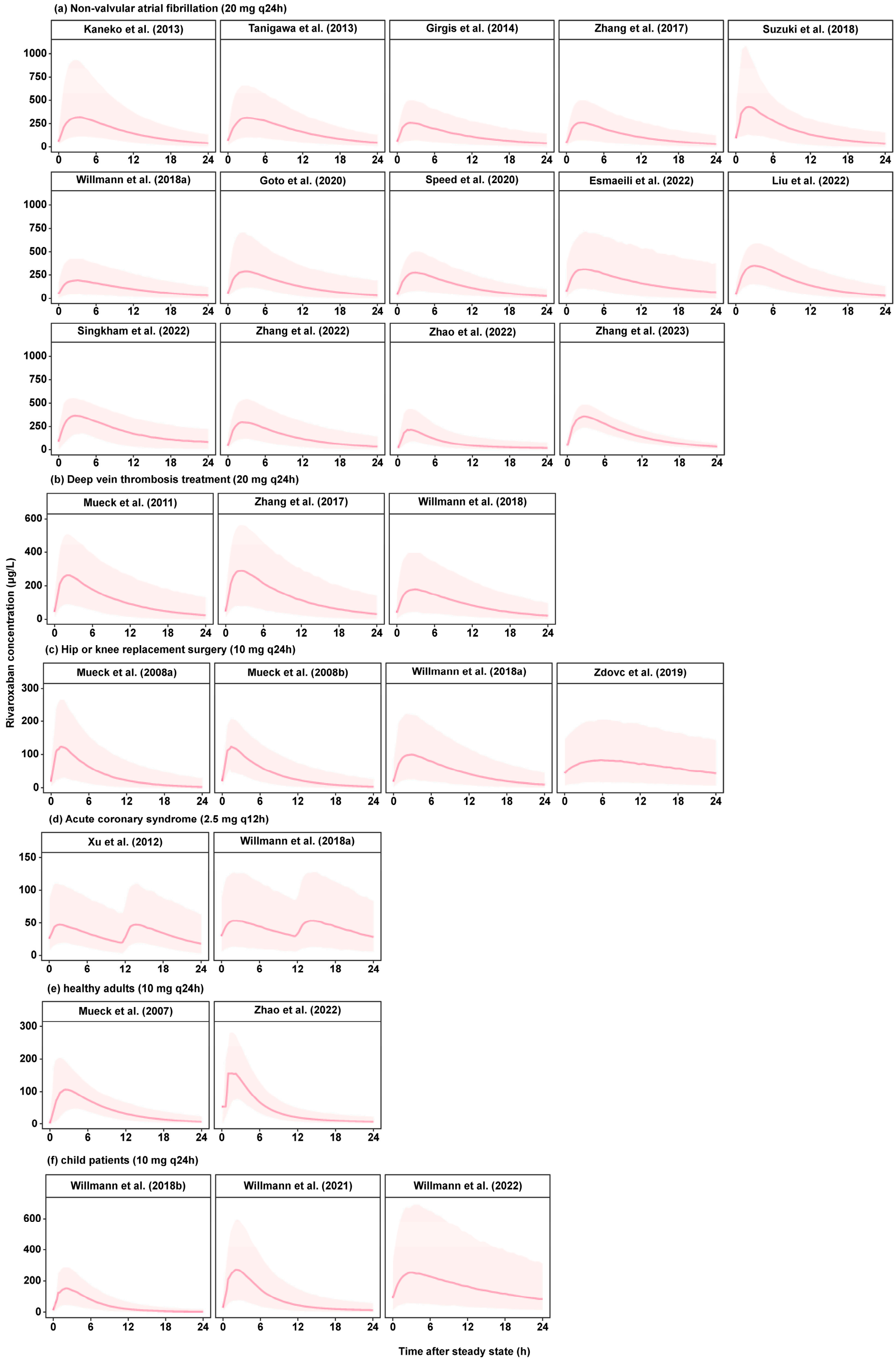

Figure S3. Concentration-time profiles of rivaroxaban at steady state.

(a) Adult patients with non-valvular atrial fibrillation; (b) adult patients treated for deep vein thrombosis and pulmonary embolism; (c) adult patients undergoing hip or knee replacement surgery; (d) adult patients with acute coronary syndrome; (e) adult healthy volunteers; (f) child patients. The typical patients were male and had the following

parameters: age, 60 years; body weight, 70 kg; serum creatinine, 1 mg/dL; lean body mass (LBM), 50 kg; creatinine clearance (CrCl, or estimated glomerular filtration rate [eGFR]), 80 mL/min; CrCl calculated using LBM, 60 mL/min; blood urea nitrogen, 16 mg/dL; hematocrit, 40%; albumin, 4 g/dL; alanine aminotransferase, 22 IU/L; total bilirubin, 14 μmol/L; transformed Child-Turcotte-Pugh Score, 5.7; *ABCB1* expression, 1.25; *ABCB1* rs1045642, TT; *ABCB1* rs4728709, GG; and without the co-administration of CYP3A4 inducers/inhibitors or P-gp inhibitors. The solid red lines represent the median of the simulated concentration-time profiles and the light pink shadows represent the 5th–95th percentiles of the concentration-time profiles.

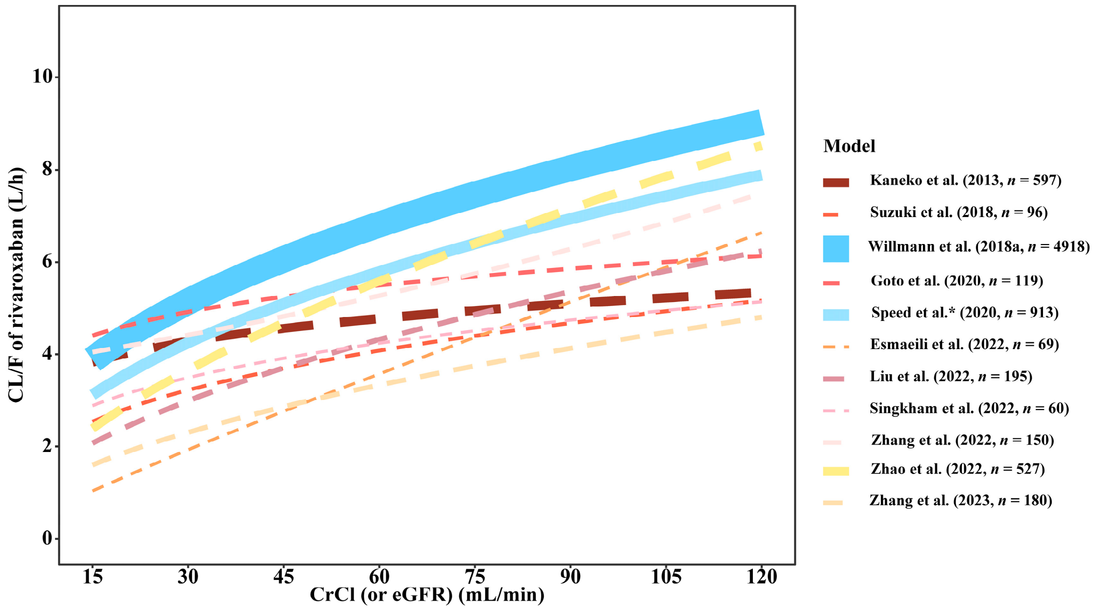

Figure S4. The apparent clearance of rivaroxaban versus renal function as reported in population pharmacokinetic models.

The models developed in the Caucasian population are shown as blue lines, and the other models were from Asian populations. The sizes of the lines correlated with the sample size in each model.

*The CrCl values in the model by Speed et al. were calculated using the lean body mass rather than total body weight.

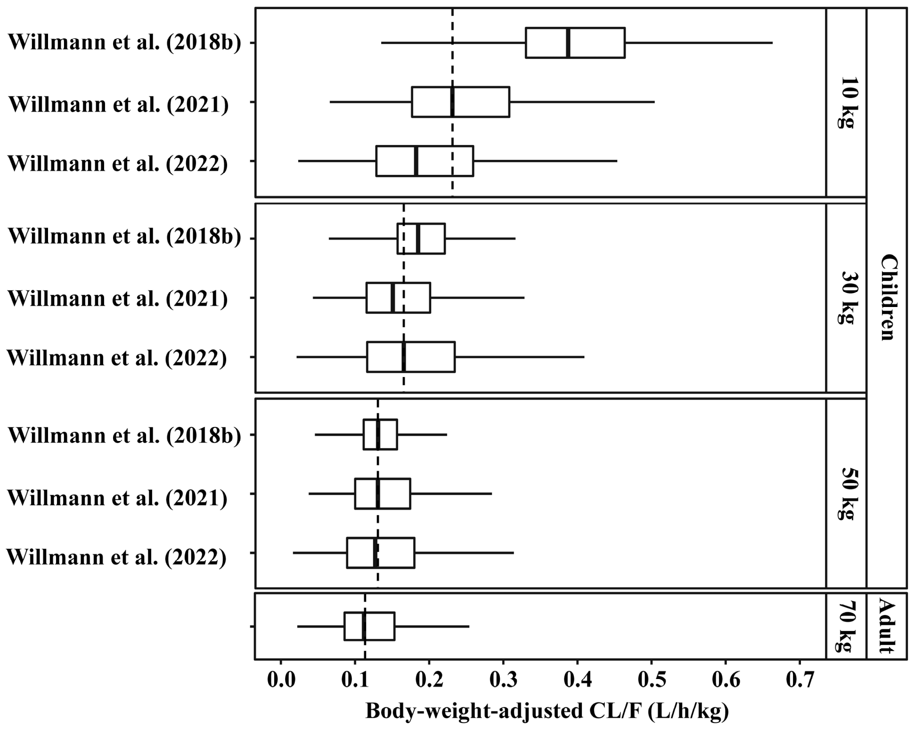

Figure S5. The apparent clearance per body weight in pediatric and adult patients.

The children were 10 years old, weighed 30 kg, and took 10 mg of rivaroxaban qd. The adults were 60 years old, weighed 70 kg, and took 20 mg of rivaroxaban qd. The bold black lines in each box represent the median body-weight-adjusted CL/F ratio in each model. The dashed lines in each panel represent the median body weight-adjusted CL/F of each population, while the dotted lines in each panel represent the 5th and 95th percentiles of the body weight-adjusted CL/F of each population.

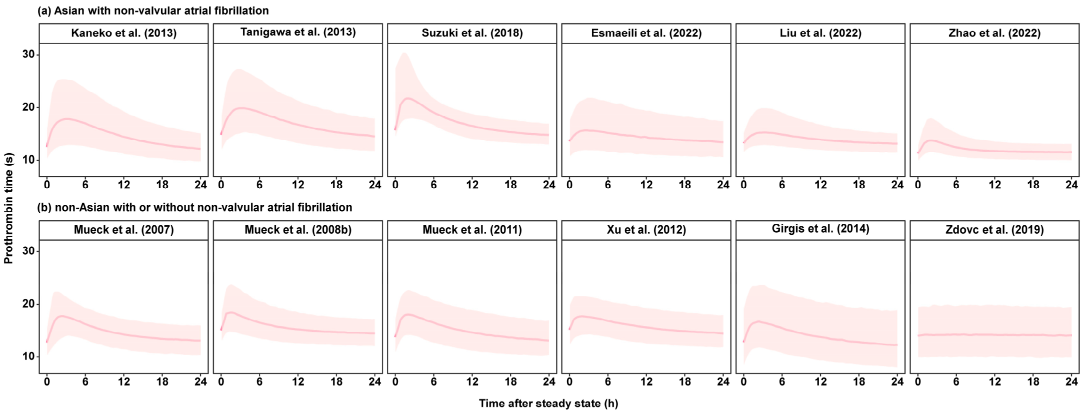

Figure S6. Prothrombin time-time profiles of rivaroxaban at steady state for (a) Asian patients with non-valvular atrial fibrillation (NVAF) and (b) Caucasian patients with or without NVAF.

The demographics of patients were male, age, 60 years; body weight, 70 kg; lean body mass, 50 kg; serum creatinine, 1 mg/dL; creatinine clearance (CrCl, or estimated glomerular filtration rate [eGFR]), 80 mL/min; CrCl calculated using LBM, 60 mL/min; blood urea nitrogen, 16 mg/dL; hematocrit, 40%; hemoglobin, 14 g/dL; albumin, 4 g/dL; alanine aminotransferase, 22 IU/L; total bilirubin, 14 μmol/L; transformed Child-Turcotte-Pugh Score, 5.7; total cholesterol, 4 mmol/L; *ABCB1* expression, 1.25; *ABCB1* rs1045642, TT; *ABCB1* rs4728709, GG; and without co-administration of CYP3A4 inducers/inhibitors or P-gp inhibitors. All patients were assumed to consume 10 mg of rivaroxaban qd with meals. The solid red lines represent the median of the simulated PT-time profiles and the light pink shadows represent the 5th-95th percentiles of the PT-time profiles.

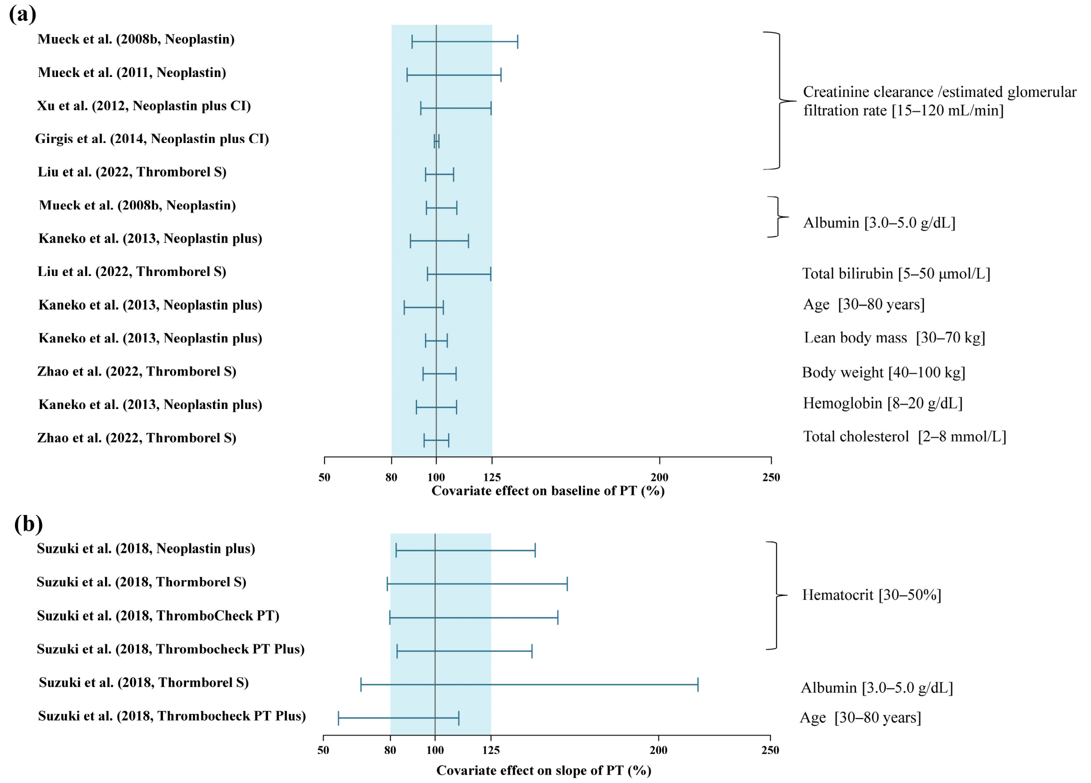

Figure S7. Effect of covariates on baseline PT (a) and slope (b).

The horizontal bars represent the effect of each covariate on the baseline PT (PT_0) or the change in PT per unit rivaroxaban concentration change (slope) in each study. The effect of each covariate on PT_0 (or slope) was characterized as the ratio of PT_0 (or slope) in the range of each covariate to the typical PT_0 (or slope).

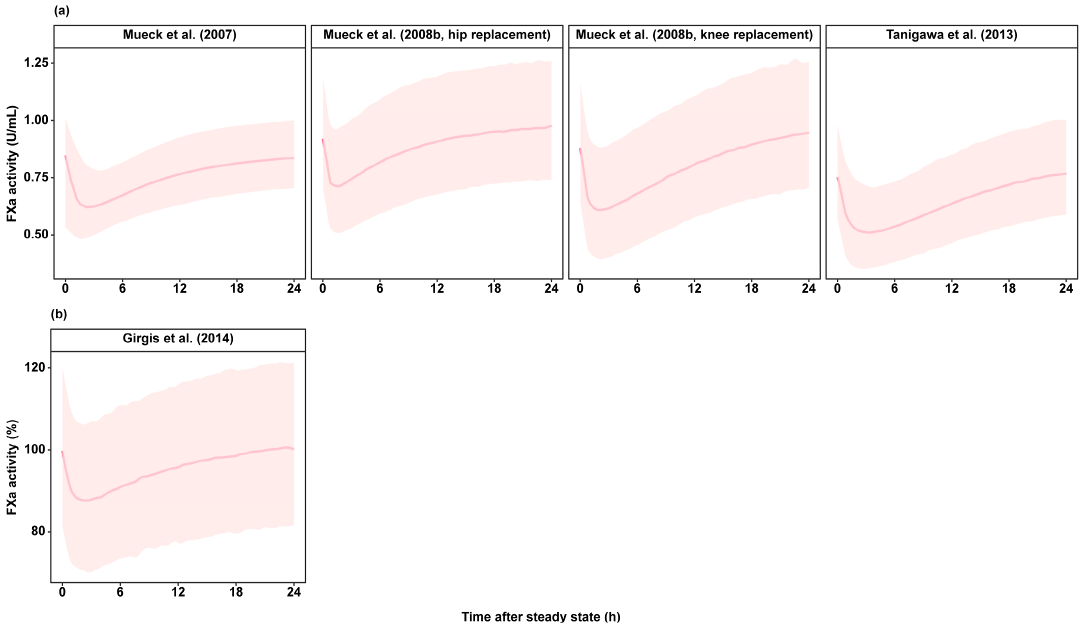

Figure S8. FXa activity-time profiles of rivaroxaban at steady state.

(a) The unit of FXa activity was expressed as U/mL. (b) FXa activity was expressed as the percentage of FXa activity in the control plasma. All patients were assumed to take 10 mg of rivaroxaban qd with meals. The solid red lines represent the median of the simulated FXa activity-time profiles and the light pink shadows represent the 5th-95th percentiles of the FXa activity-time profiles.
