# Supplementary material for: Is a Lower Dose of Rivaroxaban Required for Asians? A Systematic Review of a Population Pharmacokinetics and Pharmacodynamics Analysis of Rivaroxaban
Source: Pharmaceutics. 2023 Feb 9;15(2):588. doi: 10.3390/pharmaceutics15020588 (PMC9964148; doi:10.3390/pharmaceutics15020588)
Supplement: Supplementary file 1 [file pharmaceutics-15-00588-s001.zip › supplementary Table S2.pdf]

Table S2 Final population pharmacodynamic parameters of the included studies

Study (year)	Analytical method	Formula	Fixed effect parameters		Between-subject variability	Residual unexplained variability
**aPTT**						
Mueck et al. (2007)[1]	kaolin-activated test	$aPPT = aPTT_0 \times (1 - \frac{E_{max} \times C_p}{EC_{50} + Cp})$	$aPPT_0$	31	6.7%	11.2 s
			E_{max} (s)	42.6	/	
			EC_{50} (μg/L)	497	15.3%	
			slope (s/(μg/L))	0.0467	/	
Tanigawa et al. (2013)[2]	kaolin-activated test	$aPTT = aPTT_0 + slope \times C_p^{1-Hill \times Cp}$	$aPTT_0$	32.6	9.6%	9.1%
			slope	0.0658	31.8%	
			Hill	0.000156	/	
Zdovc et al. (2019)[3]	Pathromtin® SL	$aPPT = aPPT_0 + slope \times C_p$	$aPTT_0$ (s)	32.9	16.5%	13.1%
			slope (s/(μg/L))	2.02	/	
Esmaeili et al. (2022)[4]	Fisherbrand™	$aPPT = aPPT_0 + slope \times C_p$	$aPTT_0$ (s)	35.0	15%	13%
			slope (s/(μg/L))	0.033	28%	
**Heptest**						
Mueck et al. (2007)[1]	Haemachem	$Heptest = Heptest_0 \times (1 - \frac{E_{max} \times C_p}{EC_{50} + Cp})$	$Heptest_0$ (s)	16.2	5.5%	2.3 s
			E_{max} (s)	64	/	
			EC_{50} (μg/L)	441	7.7%	
Tanigawa et al. (2013)[2]	NA	$Heptest = Heptest_0 \times (1 - \frac{E_{max} \times C_p^{hill}}{EC_{50} + C_p^{hill}})$	$Heptest_0$ (s)	17.9	13.8%	7.0%
			E_{max} (s)	43.2	/	
			EC_{50} (μg/L)	240×[1+0.147×(ALB-4.28)]	19.64%	
			Hill	1.18	/	
**Anti-Xa activity**						
Zhao et al. (2022)[5]	Biophen DiXal	$Anti\text{-}Xa = slope \times C_p^{Hill}$	slope	0.513×1.116 (if postprandial status) (fixed[#])	11.0% (fixed[#])	22.0%
			Hill	1.10 (fixed[#])	/	12.0 μg/L
Esmaeili et al. (2022)[4]	STA®-Liquid Anti-Xa	$Anti\text{-}Xa = \frac{E_{max} \times C_p^{hill}}{EC_{50}^{hill} + C_p^{hill}}$	E_{max} (IU/mL)	4	/	31%
			EC_{50} (μg/L)	180	24%	
			Hill	1.44	108%	
**PiCT**						

Girgis et al. (2014)[6]	Pefakit® PiCT® kit	$PiCT = PiCT_0 + slope \times C_p^{1-Hill \times C_p}$	$PiCT_0$ (s)	7.97×[1-0.0016×(CrCl-76)]	46.2%	22.1%
			slope (s/(μg/L))	0.0954	5.56%	
			Hill	0.000263×[1+0.00293×(CrCl-76)]	/	

Abbreviations: ALB: albumin; aPTT: activated partial thromboplastin time; $aPTT_0$: baseline of aPTT; C_p: rivaroxaban plasma concentration; CrCl: creatinine clearance; EC_{50}: concentration generating 50% of the maximum effect; E_{max}: the maximum effect; PiCT: prothrombinase-induced clotting time; $PiCT_0$: baseline of PiCT; TBIL: total bilirubin.

Fixed to estimates from model of healthy volunteers.

Reference:

1. Mueck, W.; Becka, M.; Kubitza, D.; Voith, B.;Zuehlsdorf, M. Population model of the pharmacokinetics and pharmacodynamics of rivaroxaban - An oral, direct Factor Xa inhibitor - In healthy subjects. *Int. J. Clin. Pharmacol. Ther.* **2007,** *45*, 335-344.
2. Tanigawa, T.; Kaneko, M.; Hashizume, K.; Kajikawa, M.; Ueda, H.; Tajiri, M.; Paolini, J. F.;Mueck, W. Model-based dose selection for phase III rivaroxaban study in Japanese patients with non-valvular atrial fibrillation. *Drug Metab. Pharmacokinet.* **2013,** *28*, 59-70.
3. Zdovc, J.; Petre, M.; Pišlar, M.; Repnik, K.; Mrhar, A.; Vogrin, M.; Potočnik, U.;Grabnar, I. Downregulation of ABCB1 gene in patients with total hip or knee arthroplasty influences pharmacokinetics of rivaroxaban: a population pharmacokinetic-pharmacodynamic study. *Eur. J. Clin. Pharmacol.* **2019,** *75*, 817-824.
4. Esmaeili, T.; Rezaee, M.; Abdar Esfahani, M.; Davoudian, A.; Omidfar, D.;Rezaee, S. Rivaroxaban population pharmacokinetic and pharmacodynamic modeling in Iranian patients. *J. Clin. Pharm. Ther.* **2022,** *47*, 1284-1292.
5. Zhao, N.; Liu, Z.; Xie, Q.; Wang, Z.; Sun, Z.; Xiang, Q.;Cui, Y. A Combined Pharmacometrics Analysis of Biomarker Distribution Under Treatment With Standard- or Low-Dose Rivaroxaban in Real-World Chinese Patients With Nonvalvular Atrial Fibrillation. *Front. Pharmacol.* **2022,** *13*, 814724.
6. Girgis, I. G.; Patel, M. R.; Peters, G. R.; Moore, K. T.; Mahaffey, K. W.; Nessel, C. C.; Halperin, J. L.; Califf, R. M.; Fox, K. A.;Becker, R. C. Population pharmacokinetics and pharmacodynamics of rivaroxaban in patients with non-valvular atrial fibrillation: results from ROCKET AF. *J. Clin. Pharmacol.* **2014,** *54*, 917-27.
