# Supplementary material for: Is a Lower Dose of Rivaroxaban Required for Asians? A Systematic Review of a Population Pharmacokinetics and Pharmacodynamics Analysis of Rivaroxaban
Source: Pharmaceutics. 2023 Feb 9;15(2):588. doi: 10.3390/pharmaceutics15020588 (PMC9964148; doi:10.3390/pharmaceutics15020588)
Supplement: Supplementary file 1 [file pharmaceutics-15-00588-s001.zip › supplementary Table S3.pdf]

Table S3 Demographic information of simulated patients

	median	min	max
Adults			
Age (years)	60	30	80
Body weight (kg)	70	40	100
Lean body weight (kg)	50	30	70
Body surface area (m^2)	1.7	1.4	2.0
Body mass index (kg/m^2)	24	18	30
Gender	Male		
Serum creatinine (mg/dL)	1	0.3	2
Creatinine clearance (mL/min)	80	15	120
Creatinine clearance calculated using lean body weight (mL/min)	60	10	90
Estimated glomerular filtration rate (mL/min)	80	15	120
Blood urea nitrogen (mg/dL)	16	10	40
Hematocrit (%)	40	30	50
Albumin (g/dL)	4	2	6
Hemoglobin (g/dL)	14	8	20
Alanine aminotransferase (U/L)	22	5	40
Total bilirubin (μmol/L)	14	5	50
transformed Child-Turcotte-Pugh Score	5.7	5	8
Total cholesterol (mmol/L)	4	2	8
ABC expression	1.25	0.2	1.6
ABCB1 rs4728709	GG		
ABCB1 rs1045642	TT		
CYP3A4 inhibitor/CYP3A4 inducers/P-gp inhibitors	No		
Children			
Body weight (kg)	30	10	50
Formula	Tablet		
